# Consistent and contrasting properties of lineage-specific genes in the apicomplexan parasites *Plasmodium *and *Theileria*

**DOI:** 10.1186/1471-2148-8-108

**Published:** 2008-04-11

**Authors:** Chih-Horng Kuo, Jessica C Kissinger

**Affiliations:** 1 Department of Genetics, University of Georgia, Athens, GA 30602, USA; 2 Center for Tropical and Emerging Global Diseases, University of Georgia, Athens, GA 30602, USA; 3 Institute of Bioinformatics, University of Georgia, Athens, GA 30602, USA

## Abstract

**Background:**

Lineage-specific genes, the genes that are restricted to a limited subset of related organisms, may be important in adaptation. In parasitic organisms, lineage-specific gene products are possible targets for vaccine development or therapeutics when these genes are absent from the host genome.

**Results:**

In this study, we utilized comparative approaches based on a phylogenetic framework to characterize lineage-specific genes in the parasitic protozoan phylum Apicomplexa. Genes from species in two major apicomplexan genera, *Plasmodium *and *Theileria*, were categorized into six levels of lineage specificity based on a nine-species phylogeny. In both genera, lineage-specific genes tend to have a higher level of sequence divergence among sister species. In addition, species-specific genes possess a strong codon usage bias compared to other genes in the genome. We found that a large number of genus- or species-specific genes are putative surface antigens that may be involved in host-parasite interactions. Interestingly, the two parasite lineages exhibit several notable differences. In *Plasmodium*, the (G + C) content at the third codon position increases with lineage specificity while *Theileria *shows the opposite trend. Surface antigens in *Plasmodium *are species-specific and mainly located in sub-telomeric regions. In contrast, surface antigens in *Theileria *are conserved at the genus level and distributed across the entire lengths of chromosomes.

**Conclusion:**

Our results provide further support for the model that gene duplication followed by rapid divergence is a major mechanism for generating lineage-specific genes. The result that many lineage-specific genes are putative surface antigens supports the hypothesis that lineage-specific genes could be important in parasite adaptation. The contrasting properties between the lineage-specific genes in two major apicomplexan genera indicate that the mechanisms of generating lineage-specific genes and the subsequent evolutionary fates can differ between related parasite lineages. Future studies that focus on improving functional annotation of parasite genomes and collection of genetic variation data at within- and between-species levels will be important in facilitating our understanding of parasite adaptation and natural selection.

## Background

Comparative genomics has revealed pronounced differences in gene content across species [1]. In an early analysis of eight microbial genomes, 20–56% of the genes in a genome were shown to not have high similarity to any sequence in public databases [2]. Initially these genes were referred to as orphan genes, or ORFans, because they correspond to stretches of open reading frame in bacterial genomes that have no known relationship to other sequences. As more eukaryote genome sequences become available, the term 'lineage-specific gene' is gaining in popularity because one can specify the 'lineage specificity' of a gene to describe its phylogenetic distribution [3].

Newly evolved genes may be important for adaptation and generation of diversity [4]. For example, the protozoan parasite *Cryptosporidium parvum *possesses a set of nucleotide salvage genes that are unique among all apicomplexans surveyed to date [5]. Acquisition of the nucleotide salvage pathway from a proteobacterial source as well as other sources apparently facilitated loss of genes involved in *de novo *pyrimidine biosynthesis, rendering this parasite entirely dependent on the host for both its purines and pyrimidines. Characterization of these lineage-specific genes not only leads to a better understanding of the parasite's biology but also provides a promising therapeutic target against an important parasite, since blocking the nucleotide salvage pathway can inhibit parasite growth but not harm its human host [5].

Currently, there are several hypotheses regarding the origin of lineage-specific genes. The first model invokes the process of horizontal gene transfer, in which organisms acquire genes from other distantly related species. This mechanism can create lineage-specific genes that are not shared by closely related organisms, as in the example of nucleotide salvage enzymes in *C. parvum *[5]. Previous studies have shown that horizontal gene transfer is an important force for genome evolution in bacteria [6-8], unicellular eukaryotes [9], and multicellular eukaryotes [10].

The second model is based on gene duplication followed by rapid sequence divergence [11,12]. Based on the observation that the sequence divergence rate is positively correlated with lineage specificity in a diverse set of organisms [3,11-14], Alba and Castresana [12] proposed that newly duplicated genes may be released from selective constraint and accumulate mutations at a faster rate. While most of the mutations may be deleterious and lead to loss of function in one copy [15], it is also possible that one of the copies can acquire new functions and become a novel gene in the genome. However, whether gene duplication followed by rapid divergence is truly an important mechanism of generating lineage-specific genes is still under debate. Elhaik *et al*. [16] suggested that the correlation between divergence rate and lineage specificity may simply be an artifact, stemming from our inability to identify homologs of fast-evolving genes across distantly related taxa based on sequence similarity searches. However, a recent simulation study by Alba and Castresana [17] demonstrated that sequence similarity searches performed at the amino acid level can reliably detect fast-evolving genes due to the rate heterogeneity among sites.

In addition to the two main models discussed above, other explanations for the origin of lineage-specific genes such as *de novo *creation from non-coding sequences [18,19], exon-shuffling [20,21], intracellular gene transfer between organellar and nuclear genomes [9], and differential gene loss [22] also have been proposed. However, the relative importance of various forces that generate lineage-specific genes remains largely unknown.

While erroneous annotation has also been proposed as one explanation for the abundance of lineage-specific genes [23,24], expression data [25,26] and nucleotide substitution patterns [24,27] suggest that many lineage-specific genes are indeed functional and not annotation artifacts. Unfortunately, understanding the biological function of these genes is difficult due to the lack of homologs in model organisms to use for functional characterization. As a result, a large percentage of the lineage-specific genes that have been identified to date are annotated as hypothetical proteins of unknown function.

In this study, we aim to characterize the lineage-specific genes in a group of unicellular eukaryotes from the phylum Apicomplexa, including several important pathogens of humans and animals. The most infamous member of this phylum is the causative agent of malaria, *Plasmodium*, which causes more than one million human deaths per year globally [28]. Other important lineages include *Cryptosporidium *that causes cryptosporidiosis in humans and animals [29,30], *Theileria *that causes tropical theileriosis and East Coast fever in cattle [31,32], and *Toxoplasma *that causes toxoplasmosis in immunocompromised patients and congenitally infected fetuses [33]. The availability of genome sequences from these apicomplexan species has provided us with new and exciting opportunities to study their genome evolution. Improved knowledge of the lineage-specific genes in these important parasites can lead to a better understanding of their adaptation history and possibly identification of novel therapeutic targets.

## Results

### Inference of the species tree

**Figure 1:**
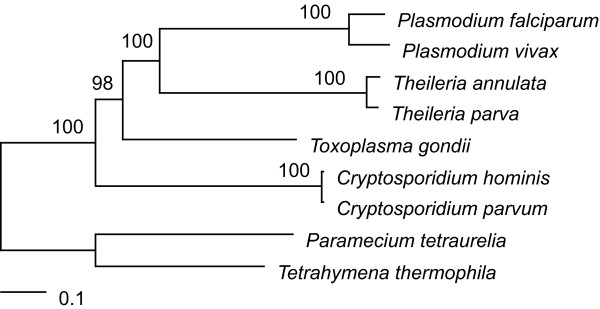
**The apicomplexan species tree**. Maximum likelihood tree generated from the concatenated alignment of 83 single-copy genes (24,494 aligned amino acid sites). Two free-living ciliates, *Paramecium tetraurelia *and *Tetrahymena thermophila*, are included as the outgroup to root the tree. Labels above branches indicate the level of clade support inferred by 100 bootstrap replicates.

We based our comparative analyses on a phylogenetic framework in order to infer the lineage specificity of individual genes. Among the nine species included in the data set (seven apicomplexans as well as two outgroup ciliates), we identified 83 single-copy genes that contain at least 100 alignable amino acid sites to infer the species tree (see Methods for details; a list of these 83 genes is provided in Additional file 1). Based on the concatenated alignment of these 83 genes (with 24,494 aligned amino acids sites), we infer a species tree with strong bootstrap support (Figure 1). This tree is consistent with our prior understanding of apicomplexan relationships based on morphology and development [34], rDNA analyses [35,36], and multigene phylogenies [37,38].

### Phylogenetic distribution of orthologous genes

**Figure 2:**
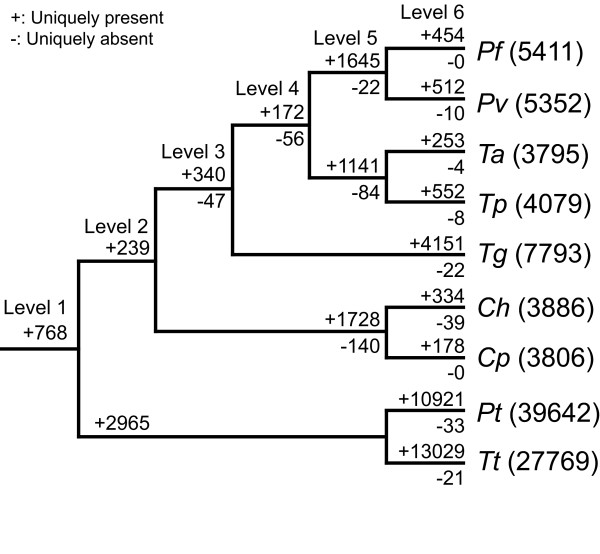
**Phylogenetic distribution of orthologous gene clusters**. The numbers after species name abbreviation (see Table 1) indicate the total number of annotated protein coding genes in the genome. The numbers above a branch and proceeded by a '+' sign indicate the number of orthologous gene clusters that are uniquely present in all daughter lineages; the numbers below a branch and proceeded by a '-' sign indicate the number of orthologous gene clusters that are uniquely absent. For example, on the internal branch that leads to the two *Plasmodium *species, 1,645 gene clusters contain sequences from both *Pf *and *Pv *but not any other species present on the tree. Similarly, there are 22 gene clusters that contain sequences from all species except *Pf *and *Pv*. Note that a gene cluster may contain more than one sequence from a species if paralogs are present in the genome. The levels refer to the degree of lineage specificity; genes in level 1 are shared by all species on the tree and genes in level 6 are species-specific.

Using the species tree (Figure 1) as the foundation, we characterized the phylogenetic distribution of orthologous gene clusters among the apicomplexan genomes analyzed (Figure 2, Table 1). The orthologous gene identification was performed using OrthoMCL [39] based on sequence similarity searches with an additional step of Markov Clustering [40] to improve sensitivity and specificity (see Methods for details). Our results indicated that many genes are genus-specific, ranging from approximately 30% of the genes in *Plasmodium *and *Theileria *up to about 45% in *Cryptosporidium*.

**Table 1:** List of species name abbreviation and data sources

Abbr.	Species name	Number of sequences	Version date	Data source
*Ch*	*Cryptosporidium hominis *[30]	3,886	12/15/2006	CryptoDB [76]
*Cp*	*Cryptosporidium parvum *[29]	3,806	04/02/2006	CryptoDB [76]
*Pf*	*Plasmodium falciparum *[28]	5,411	12/07/2005	PlasmoDB [64]
*Pv*	*Plasmodium vivax*	5,352	12/07/2005	PlasmoDB [64]
*Ta*	*Theileria annulata *[31]	3,795	07/15/2005	GeneDB [77]
*Tp*	*Theileria parva *[32]	4,079	08/30/2005	J. Craig Venter Institute [78]
*Tg*	*Toxoplasma gondii*	7,793	01/04/2006	ToxoDB [79]
*Pt*	*Paramecium tetraurelia *[80]	39,642	12/11/2006	ParameciumDB [81]
*Tt*	*Tetrahymena thermophila *[82]	27,769	04/14/2006	J. Craig Venter Institute [78]

The annotated protein sequences for each genome were downloaded from the respective data source with the version date as indicated. Two ciliates, *Paramecium tetraurelia *and *Tetrahymena thermophila*, are included as outgroups.

We selected *Plasmodium falciparum *and *Theileria annulata *for further investigations of lineage-specific genes. The asymmetrical topology of the species tree allows categorization of the genes in these two species into six levels of lineage specificity (Figure 2), yielding the highest resolution in determining the lineage specificity of a gene. The least specific genes at level 1, denoted as Pf1 for those in the *P. falciparum *genome and Ta1 for those in the *T. annulata *genome, are shared by all nine species analyzed, including two free-living ciliates; the most specific genes at level 6, denoted as Pf6 for those in the *P. falciparum *genome and Ta6 for those in the *T. annulata *genome, are species-specific. Together these six sets of genes account for 77% of annotated *P. falciparum *proteins (4,141/5,411) and 84% of annotated *T. annulata *proteins (3,191/3,795). Genes that are shared by a non-monophyletic group (e.g., shared by *P. falciparum *and *T. annulata *but are not found in any other species) are omitted from the following analyses. Additionally, the two species pairs, *P. falciparum-P. vivax *and *T. annulata-T. parva*, may have comparable divergence times in the range of approximately 80–100 million years [41,42] such that we can directly compare the properties of their species-specific genes. Finally, within the two focal genera, *P. falciparum *and *T. annulata *have a higher level of completeness of genome assembly than their sister species and thus are better choices for determining the chromosomal location of the lineage-specific genes.

### Sequence divergence

**Figure 3:**
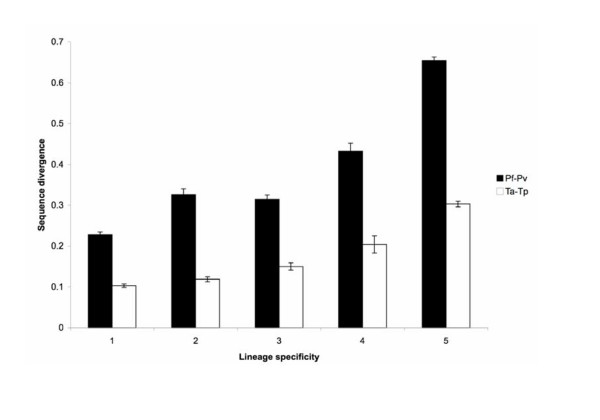
**Level of amino acid sequence divergence**. The five categories on the X-axis refer to the level of lineage specificity defined in Figure 2. Level 6 genes are not included because they are species-specific and have no orthologous sequence for comparison. Error bars indicate standard errors.

The two *Plasmodium *species, *P. falciparum *and *P. vivax*, differ greatly in their base composition. In the coding region, *P. falciparum *has a (G + C) content of 24% while *P. vivax *has a (G + C) content of 46%. Estimates of *d*_*N *_(the number of nonsynonymous substitutions per nonsynonymous site) and *d*_*S *_(the number of synonymous substitutions per synonymous site) are not reliable due to the extreme AT-bias in the *P. falciparum *genome. The average *d*_*S *_calculated from 4,159 *P. falciparum*-*P. vivax *sequence pairs is 45.7. For this reason, we quantified sequence divergence at the amino acid level based on the protein distance calculated by TREE-PUZZLE [43]. We found that the level of sequence divergence between sister taxa is positively correlated with the lineage specificity of a gene (Figure 3). The same trend is observed in both species-pairs. Compared to the two *Plasmodium *species, the *Theileria *species-pair has a lower level of sequence divergence. Level 6 genes are not included in the sequence divergence result because they are species-specific and have no orthologous sequence in the sister species for comparison.

We identified 1,701 genes that are single copy in both *Theileria *species and are reasonably conserved for substitution rate analysis at the nucleotide level (i.e., *d*_*S *_<= 1). Consistent with the sequence divergence measured at the amino acid level, nucleotide substitution rates are higher in genes with higher lineage specificity (Table 2). We do not find strong evidence of any gene under positive selection (i.e., *d*_*N*_/*d*_*S *_ratio > 1, data not shown).

**Table 2:** Nucleotide substitution rates in *Theileria*

Gene set	Number of sequences		*d*_*N*_		*d*_*S*_		*d*_*N*_/*d*_*S *_ratio	
	
	Included	Excluded	Mean	Std. Dev.	Mean	Std. Dev.	Mean	Std. Dev.
Ta1	518	299	0.05	0.04	0.69	0.15	0.07	0.05
Ta2	159	83	0.06	0.04	0.70	0.15	0.09	0.05
Ta3	227	119	0.08	0.04	0.71	0.14	0.11	0.06
Ta4	107	68	0.09	0.05	0.71	0.15	0.13	0.06
Ta5	687	593	0.13	0.07	0.73	0.14	0.19	0.10

Genes that are not single-copy or have a high level of divergence (i.e., *d*_*S *_> 1) are excluded because the substitution rate estimates are not reliable. Level 6 genes are not included because they are species-specific and have no orthologous sequence for comparison. The nonsynonymous substitution rate (*d*_*N*_) indicates the number of nonsynonymous substitutions per nonsynonymous site; the synonymous substitution rate (*d*_*S*_) indicates the number of synonymous substitutions per synonymous site.

### (G + C) content and relative codon bias

**Figure 4:**
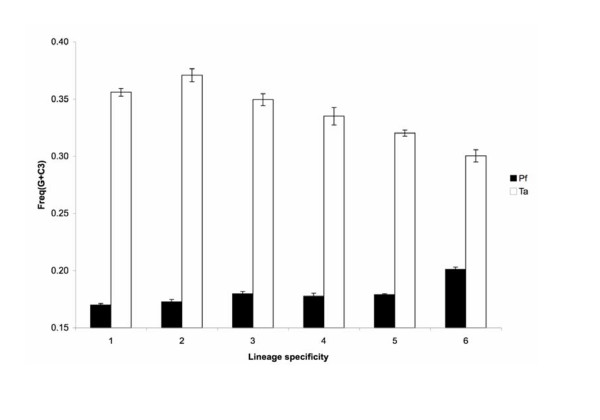
**(G + C) content at the third codon position**. The level of lineage specificity for each calculation is as defined in Figure 2. Error bars indicate standard errors.

The average (G + C) content at the third codon position (i.e., (G+C3)) increases with lineage specificity in *P. falciparum *(Figure 4), suggesting that phylogenetically conserved genes are biased toward AT-rich codons in this extremely AT-rich genome. In *T. annulata*, the opposite trend is observed; genes with high lineage specificity have a lower (G + C) content at the third codon position (Figure 4).

We used the relative codon bias developed by Karlin *et al*. [44] to compare the differences in codon usage between different gene sets within each species (Table 3). In both *P. falciparum *and *T. annulata*, the level 6 (i.e., species-specific) genes exhibit a high level of deviation with regard of their codon preference compared to the other gene sets (see Methods for details). In *P. falciparum*, the average pairwise difference in all comparisons is 0.049 and the mean pairwise difference involving Pf6 genes is 0.102 (Table 3A). In *T. annulata*, the average pairwise difference in all comparison is 0.098 and the mean pairwise difference involving Ta6 genes is 0.183 (Table 3B).

**Table 3:** Relative codon bias

A.							
	All	Pf1	Pf2	Pf3	Pf4	Pf5	Pf6

All	*	0.037	0.026	0.015	0.019	0.015	0.087
Pf1	0.040	*	0.030	0.033	0.045	0.047	0.115
Pf2	0.026	0.028	*	0.017	0.037	0.032	0.107
Pf3	0.015	0.031	0.017	*	0.027	0.020	0.102
Pf4	0.019	0.042	0.037	0.026	*	0.021	0.094
Pf5	0.014	0.042	0.031	0.019	0.020	*	0.095
Pf6	0.091	0.115	0.110	0.104	0.101	0.103	*

B.							

	All	Ta1	Ta2	Ta3	Ta4	Ta5	Ta6

All	*	0.055	0.084	0.032	0.034	0.043	0.159
Ta1	0.055	*	0.037	0.040	0.077	0.098	0.206
Ta2	0.084	0.037	*	0.064	0.105	0.127	0.231
Ta3	0.032	0.040	0.064	*	0.046	0.068	0.187
Ta4	0.034	0.078	0.106	0.047	*	0.040	0.162
Ta5	0.043	0.098	0.127	0.068	0.040	*	0.130
Ta6	0.165	0.215	0.239	0.196	0.167	0.135	*

The relative codon bias between groups of genes was calculated based on the method developed by Karlin *et al*. [44]. The gene sets listed in columns are used as the reference and the gene sets listed in rows are the focal set. A. Relative codon bias between sets of *P. falciparum *genes. B. Relative codon bias between sets of *T. annulata *genes.

### Functional analyses based on annotation

As expected, most of the phylogenetically conserved genes have functional annotation or have at least one identifiable protein domain (Table 4). As the phylogenetic distribution of a gene becomes more restricted, it is more likely to be annotated as a hypothetical protein. Functional analysis based on available gene annotation indicates that most conserved genes (levels 1 and 2) are responsible for basic cellular processes (e.g., DNA replication, transcription, translation, etc), while most genus- and species-specific genes (levels 5 and 6) are hypothetical proteins of unknown function (see Additional files 2 and 3). Despite the poor annotation of genus- and species-specific genes, 87% of level 5 genes and 72% of level 6 genes in *P. falciparum *have expression data available based on oligonucleotide microarrays [26]. This result suggests that most of the hypothetical proteins are real genes and not annotation artifacts.

**Table 4:** Characteristics of lineage-specific genes in *Plasmodium falciparum*

Gene set	Number of gene clusters	Number of *P. falciparum *genes	Average protein length (a.a.)	Frequency of genes with			
				
				"Hypothetical protein" in product description	Pfam domains	Expression data	Predicted signal peptide or transmembrane domains
Pf1	768	803	718	0.26	0.96	0.92	0.16
Pf2	239	244	998	0.70	0.84	0.91	0.29
Pf3	340	346	650	0.66	0.74	0.88	0.49
Pf4	172	175	803	0.74	0.65	0.93	0.39
Pf5	1645	1687	839	0.88	0.53	0.87	0.41
Pf6	454	886	481	0.63	0.46	0.72	0.62
Pf6A	451	616	340	0.91	0.25	0.71	0.56

Gene sets from Pf1 through Pf6 refer to the orthologous gene clusters present in the six levels of lineage specificity defined in Figure 2. Pf6A is the same as Pf6 except that it excludes three surface antigen gene families (i.e., PfEMP1, rifin, and stevor). Note that there may be more than one *P. falciparum *gene in a gene cluster when paralogous genes are present in the genome.

The two focal lineages in our analysis, *Plasmodium *and *Theileria*, exhibit one interesting difference in terms of the phylogenetic distribution of surface antigens. We found that surface antigens are species-specific in *Plasmodium *and genus-specific in *Theileria*. All members of the three large surface antigen protein families in *P. falciparum *genome, including 161 rifin, 74 PfEMP1, and 35 stevor, are found in the Pf6 list and have no ortholog in *P. vivax*. Of the 163 *T. annulata *proteins that contain FAINT, a protein domain that associates with proteins exported to the host cell [31], 116 are in the Ta5 list (i.e., shared by *T. annulata *and *T. parva*) and only 28 are in the Ta6 list (i.e., specific to *T. annulata*).

In *P. falciparum *41% of the genus-specific proteins and 62% of the species-specific proteins contain a putative signal peptide or at least one predicted transmembrane domain (Table 4), which suggests that these proteins may be exported to the host cell or present on the surface of the parasite or its vacuole. This result is consistent with the hypothesis that lineage-specific genes in apicomplexan parasites are likely to be involved in host-parasite interactions and thus, potentially adaptation.

### Chromosomal location

**Figure 5:**
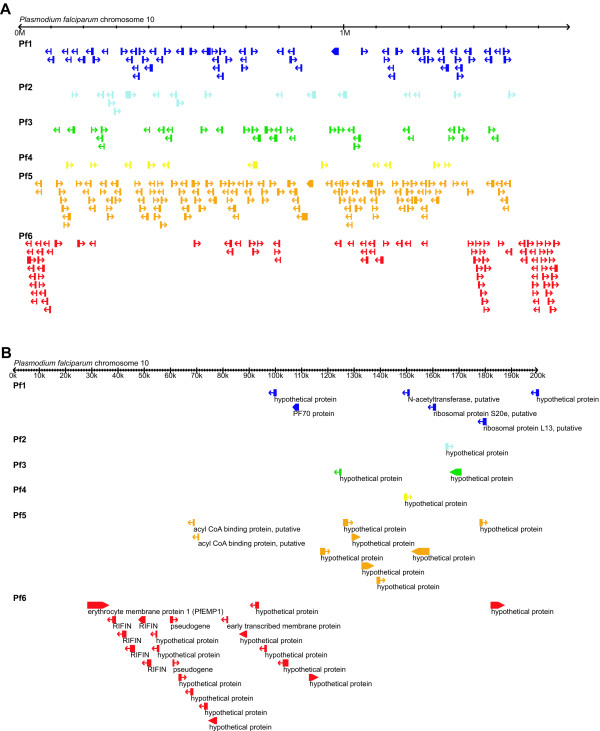
**Chromosomal location of genes in *Plasmodium falciparum***. Chromosomal location of genes on *P. falciparum *chromosome 10. See Additional file 4 for views of all 14 chromosomes in this species. The level of lineage specificity is as defined in Figure 2. A. View of entire chromosome 10 (MAL10). B. Close-up view of the first 200 kb of chromosome 10.

**Figure 6:**
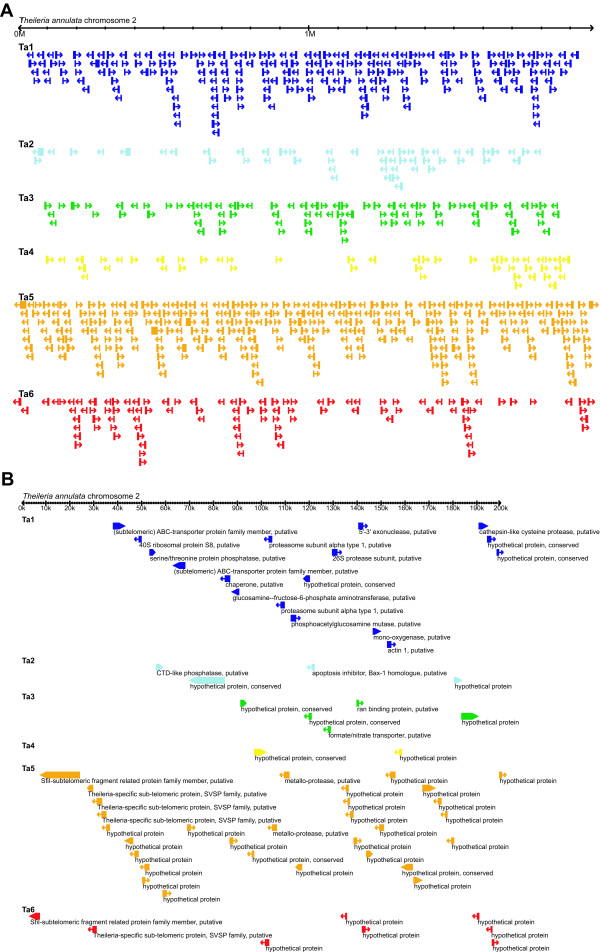
**Chromosomal location of genes in *Theileria annulata***. Chromosomal location of genes on *T. annulata *chromosome 2. See Additional file 5 for views of all four chromosomes in this species. The level of lineage specificity is as defined in Figure 2. A. View of entire chromosome 2. B. Close-up view of the first 200 kb of chromosome 2.

Analysis of chromosomal location demonstrated that most species-specific genes in *P. falciparum *are located near chromosome ends (see Figure 5 for one example chromosome and Additional file 4 for all 14 chromosomes). In *T. annulata *(see Figure 6 for one example chromosome and Additional file 5 for all four chromosomes), we observed a similar pattern that the regions adjacent to chromosome ends are devoid of the phylogenetically conserved genes (cf. Figures 5B and 6B). However, unlike the pattern found in *P. falciparum*, most of the species-specific genes in *T. annulata *(i.e., Ta6) are distributed across the entire length of chromosomes and are not enriched in the regions adjacent to chromosome ends (cf. Figures 5A and 6A).

**Figure 7:**
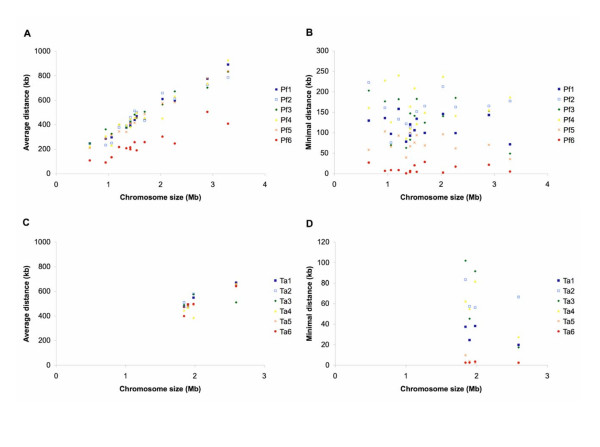
**Average and minimal distance of mapped genes to chromosome end**. The level of lineage specificity is as defined in Figure 2. A. Average distance to chromosome end in *Plasmodium falciparum*. B. Minimum distance to chromosome end in *P. falciparum*. C. Average distance to chromosome end in *Theileria annulata*. B. Minimum distance to chromosome end in *T. annulata*.

To quantify the pattern of gene distribution on chromosomes, we calculated the distance of each gene to the nearest chromosome end. For each set of genes (levels 1 through 6 in each species), we utilized (1) the average distance to the nearest chromosome end and (2) the minimal distance to the nearest chromosome end (i.e., the minimal found in a given gene set) for this analysis. In *P. falciparum*, the average distance scales with chromosome size and the species-specific genes (i.e., Pf6) are closer to chromosome ends (Figure 7A). In contrast, minimal distance does not scale with chromosome size (Figure 7B). For all chromosomes, the minimal distances of phylogenetically conserved genes from the chromosome ends (i.e., Pf1 through Pf4) are larger than 50–100 kb. This result indicates that the regions that are occupied exclusively by genus- and species-specific genes are proportionally larger in smaller chromosomes. Consistent with this observation, three of the smallest chromosomes in *P. falciparum *(i.e., MAL1, MAL2, and MAL4) have many more species-specific genes than random expectation (Chi-square test d.f. = (6 gene sets -1) * (14 chromosomes - 1) = 65, P-value = 1e-12).

In *T. annulata*, genes with different levels of lineage specificity have similar average distances to chromosome ends (Figure 7C). This result corroborates the visual pattern in Figure 6A that species-specific genes are distributed across the entire length of a chromosome, in contrast to the clustering near chromosome ends observed in *P. falciparum *(Figure 5A). For all four chromosomes in *T. annulata*, the regions that are adjacent to chromosome ends and devoid of phylogenetically conserved genes (i.e., Ta1 through Ta4) are approximately 20–40 kb (Figure 7D), a distance smaller than in *P. falciparum*. Unlike the pattern found in *P. falciparum *in which species-specific genes are closer to chromosome ends than genus-specific genes, genus- and species-specific genes in *T. annulata *(i.e., Ta5 and Ta6) have similar minimal distances in all four chromosomes (Figure 7D).

**Figure 8:**
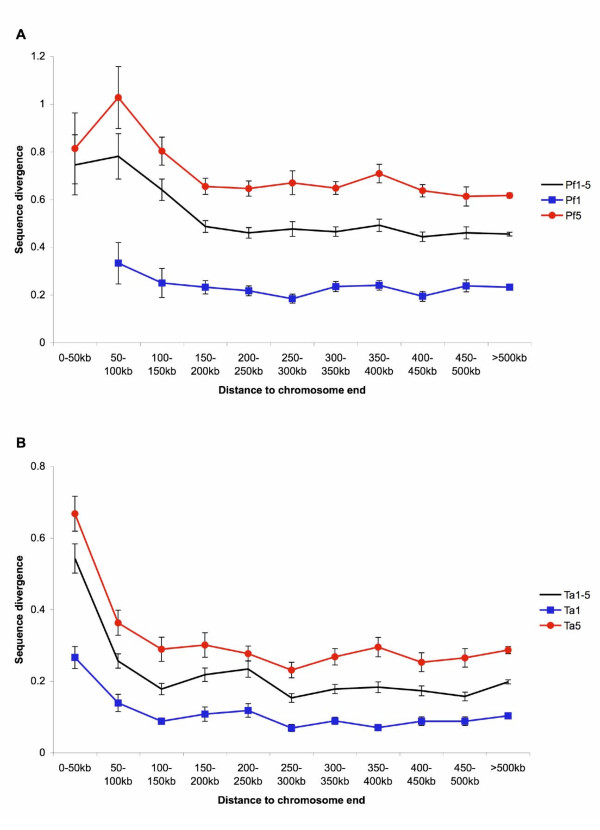
**Amino acid sequence divergence and chromosomal location**. Plot of amino acid sequence divergence as a function of the distance to the nearest chromosome end. A. *Plasmodium falciparum*. B. *Theileria annulata*. The black lines in both panels (i.e., Pf1-5 in panel A and Ta1-5 in panel B) refer to the combined results from genes with five different levels of lineage specificity and are included as the background reference. Error bars indicate standard errors.

In both *P. falciparum *and *T. annulata*, genes located near chromosome ends have a higher level of sequence divergence relative to its ortholog in the sister species at the amino acid level (Figure 8). This trend is observed in genes with different levels of lineage specificity and is stronger in *T. annulata*.

## Discussion

We identified a pattern in which lineage-specific genes have a higher level of sequence divergence among sister species in a group of important protozoan parasites. This result is consistent with previous studies in bacteria [13], fungi [3], and animals [11,12,14]. Now we further confirm that this pattern also holds true in a protistan phylum, suggesting that it may be universal across much of the tree-of-life. Results from functional analyses agree with our intuitive expectation that conserved genes are involved in basic cellular functionalities and are well annotated. A large number of the lineage-specific genes (at the species level in *Plasmodium *and the genus level in *Theileria*) are found to be putative surface antigens that the parasites use to interact with their hosts. This result supports the hypothesis that lineage-specific genes may be important in adaptation [4]. In addition, the physical distance of a gene to the nearest chromosome end is correlated with the level of sequence divergence.

We found three contrasting properties of lineage-specific genes between two major apicomplexan lineages. First, families of surface antigens are species-specific in *Plasmodium *but genus-specific in *Theileria*. Second, most of the species-specific genes are located in sub-telomeric regions in *P. falciparum *but no such pattern exists in *T. annulata*. Third, the (G + C) content at the third codon position increases with lineage specificity in *P. falciparum *but decreases in *T. annulata*. Taken together, these results suggest that the mechanisms of generating lineage-specific genes and their subsequent evolutionary fates differ between apicomplexan parasite lineages.

### Gene content evolution

All apicomplexan species analyzed have small genomes compared to the free-living out-group. This result is consistent with comparative genomic analyses conducted in other pathogenic bacteria and eukaryotes; extreme genome reduction is a common theme in the genome evolution of these organisms [45].

A large proportion of the genes in apicomplexans are genus-specific (Figure 2). One parsimonious explanation for this observation is that each lineage acquired a new set of genes during its evolutionary history. An alternative explanation invokes differential loss among lineages when evolving from a free-living ancestor with a relatively large genome. We found that 23% of the protein coding genes in *P. falciparum *and 16% in *T. annulata *have a complex phylogenetic distribution pattern and do not fit into a simple single gain/loss model. These results suggest that some ancestral genes in the apicomplexans may have experienced multiple independent losses during their evolutionary history. Further investigation is necessary to distinguish true gene gains from differential retention of ancestral genes.

### Comparison of genes with different levels of lineage specificity

Consistent with previous studies in bacteria [13], fungi [3], and animals [11,12,14], we observed a pattern in which sequence divergence is higher in genes with a higher level of lineage specificity. One explanation is that phylogenetically conserved genes are often involved in fundamental cellular processes (see Results). These genes are likely to be under purifying selection that constrains the rate of sequence divergence. In support of this hypothesis, we observe that the mean *d*_*N*_/*d*_*S *_ratio among the level 1 genes in *Theileria *is only 0.07 (Table 2), indicating an extremely low rate of nonsynonymous substitution relative to synonymous substitution.

Based on the hypothesis that lineage-specific genes are often involved in adaptation [4], such as invasion of hosts or evasion of the immune responses, lineage-specific genes may be under positive selection and have a faster rate of sequence divergence. Our data is suggestive in this regard, as genus-specific genes exhibit higher sequence divergence than genes with lower levels of lineage specificity. Unfortunately we cannot directly test the hypothesis that lineage-specific genes are more likely to be under positive selection using the *d*_*N*_/*d*_*S *_ratio data. The level of sequence divergence is too high in both species pairs for such analysis. Practically all of the genes from the *Plasmodium *pair and approximately 1,000 genes from the *Theileria *pair (i.e., more than a quarter of the gene repertoire) have a *d*_*S *_estimate that is larger than one. Under this high level of sequence divergence, we cannot confidently estimate the substitution rate due to saturation. Better detection of positive selection in these genes requires data on genetic variation at within- and between-species levels [46,47].

Codon bias analyses indicate that species-specific genes have a different codon preference compared to other genes in the same genome, whereas the genes with lower levels of lineage specificity are relatively similar to each other (Table 3). It is possible that species-specific genes are relatively young and have yet to adapt to the codon usage pattern of the genome. Support for this hypothesis provided by the observation that the (G + C) content at the third codon position is much lower in the phylogenetically conserved genes in *P. falciparum *(Figure 4), suggesting that these 'older' genes are more biased toward GC-poor codons in this AT-rich genome. Alternatively, some species-specific genes may be subject to a different pattern of selection and thus possess different codon preference.

For the lineage-specific genes at the genus and species level that have functional annotations, many are known surface antigens. Because surface antigens are used by the parasites to interact with their hosts [48], such as adhesion to the cell surface or evasion of the host immune response, this result supports the hypothesis that (at least some) lineage-specific genes are involved in host-parasite interactions and have facilitated lineage-specific adaptation. Interestingly, surface antigens are species-specific in *Plasmodium*, but are genus-specific in *Theileria*. In addition, 62% of *P. falciparum*-specific genes contain a putative signal peptide or at least one predicted transmembrane domain. This result is consistent with one previous study that compared *P. falciparum *with three other *Plasmodium *species that cause rodent malaria [49]. Of the 168 *P. falciparum*-specific genes identified in this previous study that are not located in sub-telomeric regions, 68% are predicted to be exported to the surface of the parasites or the infected host cells.

### Comparison between *Plasmodium *and *Theileria*

Previous studies suggest that the two focal species pairs have similar divergence times. The two *Plasmodium *species diverged about 80–100 million years ago [41] and the two *Theileria *species diverged about 82 million years ago [42]. Our results indicate that sequence divergence is much higher between the two *Plasmodium *species (Figures 1 and 3). This may be caused by the difference in nucleotide composition, since *P. falciparum *has a GC content of 24% while *P. vivax *has a GC content of 46% in the coding region. Bias in nucleotide composition has been shown to change codon usage and amino acid composition [50]. Alternatively, it is also possible that the divergence time between *T. annulata *and *T. parva *was overestimated because it was based on a simplified assumption that the synonymous substitution rate in *Theileria *is similar to that in *Plasmodium *[42].

In both *P. falciparum *and *T. annulata*, the sub-telomeric regions contain exclusively genus- or species-specific genes. Interestingly, the physical size of these regions is not correlated with chromosome size. This observation indicates that these regions are proportionally larger in smaller chromosomes and helps explains the pattern that the three small chromosomes in *P. falciparum *have many more species-specific genes than predicted by random expectations (see Results). In addition, genes that are located near a chromosome end have a higher level of sequence divergence in both species, regardless of their lineage specificity (Figure 8). The high evolutionary rates in sub-telomeric regions are shared by many eukaryotic lineages; high rates of inter-chromosomal recombination, local duplication, and segmental rearrangement have been reported in organisms including humans [51], yeasts [52], and plants [53].

Given the high rates of evolution in sub-telomeric regions, it may be advantageous for pathogens to have their surface antigen genes located in these evolutionary hotspots to facilitate the generation of antigenic diversity. Consistent with this hypothesis, many micro-parasites have large gene families that encode surface antigens in sub-telomeric regions (reviewed in [54]). The best-studied example is the causative agent of African trypanosomiasis, *Trypanosoma brucei*. The *vsg *gene family in *T. brucei *encodes variant surface glycoproteins (VSG) that form a dense coat on the outside of the parasite. In the bloodstream stage, *T. brucei *sequentially expresses different members of the *vsg *gene family, one at a time, to generate antigenic variation [55]. The positioning of *vsg *genes in the genome is tightly linked to regulation of expression; the actively expressed *vsg *is duplicated into one of the bloodstream expression sites located in the sub-telomeric regions (reviewed in [56,57]). This homologous recombination process which involves loci that are not positional alleles is hypothesized to be important in generating genetic diversity within the gene family [54]. Although the genes encoding surface antigens in *P. falciparum *are not known to be duplicated into specific expression sites as observed in *T. brucei*, the clustering of these genes in sub-telomeric regions can facilitate inter-chromosomal recombination that increases antigenic variation [58].

We found that most of the surface antigen genes in *P. falciparum *are located in sub-telomeric regions, as previously noted [28]. Several studies have established the importance of genome location in the generation and maintenance of antigenic variation in *P. falciparum *[58,59]. The surface antigen PfEMP1 possessed by *P. falciparum *is exported to the cell surface of infected erythrocytes. PfEMP1 can remove infected erythrocytes from blood circulation by cellular adherence to microvascular endothelial cells and avoid spleen-dependent killing [60]. The study on genetic structuring suggested that the approximately 60 copies of *var *genes (which encode PfEMP1) in the *P. falciparum *genome can be divided into three functionally diverged groups with two in sub-telomeric regions and one close to the centers of chromosomes [59]. Furthermore, the recombination rate is found to be high among members in the same functional group but low for members belonging to different groups. This recombinational hierarchy may facilitate the generation of genetic diversity within a group and promote specialization between different groups. Experimental evidence suggests that the clustering of *var *genes in the sub-telomeric regions is important in the epigenetic regulation of gene expression in *P. falciparum *[61,62].

Given the generality of association between surface antigen genes and sub-telomeric regions in micro-parasites, it is interesting to see that *T. annulata *appears to be an exception to this rule. This finding may provide an explanation for the difference in host range between the two apicomplexan lineages. Because a large percentage of surface antigen genes in *Plasmodium *are located in sub-telomeric regions, the generation of antigenic variation may be faster in *Plasmodium *than in *Theileria*. Our results indicate that gene families encoding surface antigens in *Plasmodium *are highly diverged between species within the genus, whereas the two *Theileria *species still share most of their surface antigens and the genes encoding them are distributed across the entire lengths of chromosomes. For this reason, *Plasmodium *may be able to adapt to new host species at a faster rate, resulting in its much wider host range compared to *Theileria*; *Plasmodium spp*. can infect mammals, birds, and reptiles, whereas *Theileria spp*. are limited to ruminants [34].

## Conclusion

Our results agree with previous observations in other organisms that lineage-specific genes have a higher level of sequence divergence compared to phylogenetically conserved genes. In addition, two major apicomplexan lineages may have different mechanisms for generating or retaining species-specific genes. Because many lineage-specific genes in these parasites are surface antigens that interact with the host, future investigations on genome evolution in these parasites may facilitate the identification of new therapeutic or vaccine targets. Future studies that focus on improving functional annotation of parasite genomes and the collection of genetic variation data at different phylogenetic levels will be important in our understanding of parasite adaptation and natural selection.

## Methods

### Data source and orthologous gene identification

The data sources of the annotated proteins are listed in Table 1. Protein domain identification was performed with HMMPFAM [63] (version 20.0). Transmembrane domain prediction [28] and gene expression data [26] of annotated *Plasmodium falciparum *genes were downloaded from PlasmoDB [64] (Release 5.3).

Orthologous gene clusters were identified using OrthoMCL [39] (version 1.3, April 10, 2006) with default parameter settings. The ortholog identification process in OrthoMCL is largely based on the popular criterion of reciprocal best-hits but also involves an additional step of Markov Clustering [40] to improve sensitivity and specificity. We used WU-BLAST [65] (version 2.0) for the all-against-all BLASTP similarity search step with the e-value cutoff set to 1e-15.

### Phylogenetic inference

Based on the orthologous gene clustering result, we identified genes that are shared by all nine species to infer the species tree. Orthologous gene clusters that contain more than one gene from any given species were removed to avoid the complications introduced by paralogous genes in phylogenetic inference. Of the 768 orthologous gene clusters that are shared by all nine species (Figure 2), 154 clusters were single-copy in all species. For each gene, CLUSTALW [66] (version 1.83) was used for multiple sequence alignment. We enabled the 'tossgaps' option to ignore gaps when constructing the guide tree and used the default settings for all other parameters. The alignments produced by CLUSTALW were filtered by GBLOCKS [67] (version 0.91b) to remove regions that contain gaps or are highly divergent. Individual genes that had less than 100 aligned amino acid sites (33/154) or contained identical sequences from different taxa (38/154) after GBLOCKS filtering were eliminated from further analysis. We concatenated the alignments from the remaining 83 genes (with a total of 24,494 aligned amino acid sites) and utilized PHYML [68] to infer the species tree based on the maximum likelihood method. We used PHYML to estimate the proportion of invariable sites and the gamma distribution parameter (with eight substitution categories). The substitution model was set to JTT [69] and we enabled the optimization options for tree topology, branch lengths, and rate parameters. To estimate the level of support on each internal branch, we performed 100 non-parametric bootstrap samplings.

### Quantification of sequence divergence

The nonsynonymous and synonymous substitution rates at the nucleotide level (i.e., *d*_*N *_and *d*_*S*_) were estimated using CODEML in the PAML package [70]. We performed pairwise sequence alignment at the amino acid level using CLUSTALW [66] with default parameters for all orthologous genes that are single copy in both *Plasmodium *species or both *Theileria *species. The protein alignments were converted into the corresponding nucleotide alignments using NAL2PAL [71] (version 12). All gap positions were removed from the alignments before the substitution rate estimation by CODEML. To avoid problems of inaccurate rate estimation caused by saturation, we excluded sequences with a synonymous substitution rate (*d*_*S*_) that is greater than one.

To quantify the level of sequence divergence at the amino acid level, we used TREE-PUZZLE [43] to calculate the protein distance between orthologs in sister species. The parameters were set to the JTT substitution model [69], mixed model of rate heterogeneity with one invariable and eight Gamma rate categories, and the exact and slow parameter estimation. Orthologous sequences were first aligned using CLUSTALW [66] followed by a filtering step using GBLOCKS [67] to remove gaps and highly divergent regions before the calculation of protein distance. Five sequences (PFA0650w, PFD0105c, PFL0060w, and PFD1140w from *P. falciparum *and TA18345 from *T. annulata*) that were not reliably aligned to their ortholog in the sister species were excluded from this analysis.

### Calculation of relative codon bias

The relative codon bias between sets of genes in the two focal species, *P. falciparum *and *T. annulata*, was calculated based on the method developed by Karlin et al. [44]. Briefly, the method considers two sets of genes, one focal set and one reference set, and calculates the difference in relative frequency of codon family that encode the same amino acid between the two sets. The theoretical maximum of the difference between two sets of genes is 2.000, but the empirical values based on biological data generally range from 0.050 to 0.300 [44,72,73]. This measurement is different from the conventional codon adaptation index (CAI) developed by Sharp and Li [74], in which a set of highly expressed genes is always used as the reference set. We choose the relative codon bias to measure codon preference because it can provide a better resolution under certain conditions. For example, two sets of weakly expressed genes may have similar values of codon adaptation index but still possess vastly different codon preferences.

### Visualization and quantification of chromosomal location

GBROWSE [75] was used for visualization of gene distribution on chromosomes. To quantify the pattern of chromosomal location, we calculated the distance of each gene to the nearest chromosome end. For example, the *P. falciparum *gene PF10_0023 on chromosome MAL10 (physical size is 1,694,445 bp) starts at position 99,380 and ends at 100,362. Its distance to the nearest chromosome end was calculated as 99,380 - 1 = 99,379 bp. For gene PF10_0369 on the same chromosome that starts at 1,493,991 and ends at 1,496,955, its distance to the nearest chromosome end was calculated as 1,694,445 – 1,496,955 = 197,490 bp. The orientation of a gene (i.e., whether it is on the '+' strand or the '-' strand) is ignored for distance calculation.

## Authors' contributions

CHK developed the concept of this study, performed the analysis, and wrote the manuscript. JCK provided supervision, feedback, and comments on the manuscript. All authors have read and approved the final manuscript.

## Supplementary Material

Additional file 1 Genes used to infer the species tree. List of the 83 single-copy genes used to infer the species tree. Gene ID and description are based on the *Plasmodium falciparum *sequence annotation in each ortholog group. Click here for file

Additional file 2 Lists of lineage-specific genes in *Plasmodium falciparum*. Lists of lineage-specific genes, grouped by the level of lineage specificity defined in Figure 2. Click here for file

Additional file 3 Lists of lineage-specific genes in *Theileria annulata*. Lists of lineage-specific genes, grouped by the level of lineage specificity defined in Figure 2. Click here for file

Additional file 4 Chromosomal location of lineage-specific genes in *Plasmodium falciparum*. Graphical distribution of genes on all 14 *Plasmodium falciparum *chromosomes. Click here for file

Additional file 5 Chromosomal location of lineage-specific genes in *Theileria annulata*. Graphical distribution of genes on all 4 *Theileria annulata *chromosomes. Click here for file
