# Supplementary figures and images for: Consistent and contrasting properties of lineage-specific genes in the apicomplexan parasites Plasmodium and Theileria
Source: BMC Evol Biol. 2008 Apr 11;8:108. doi: 10.1186/1471-2148-8-108 (PMC2330040; doi:10.1186/1471-2148-8-108)

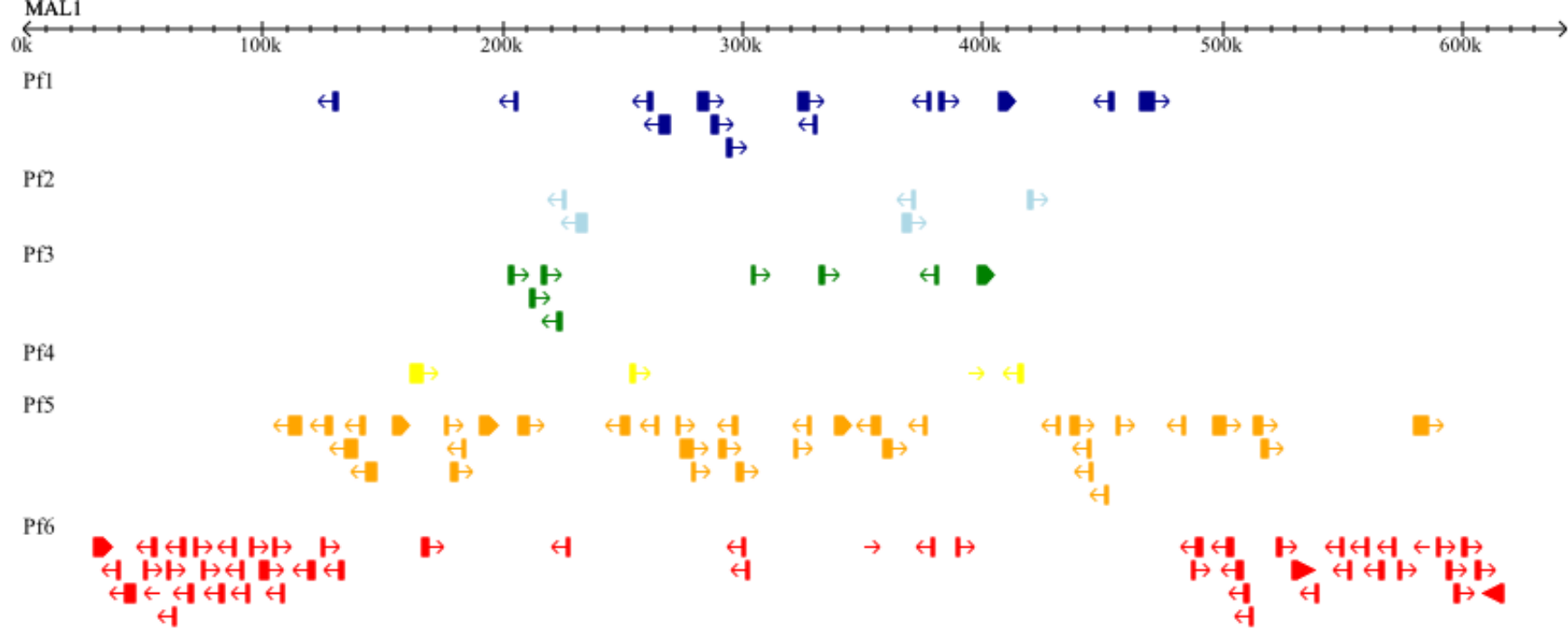

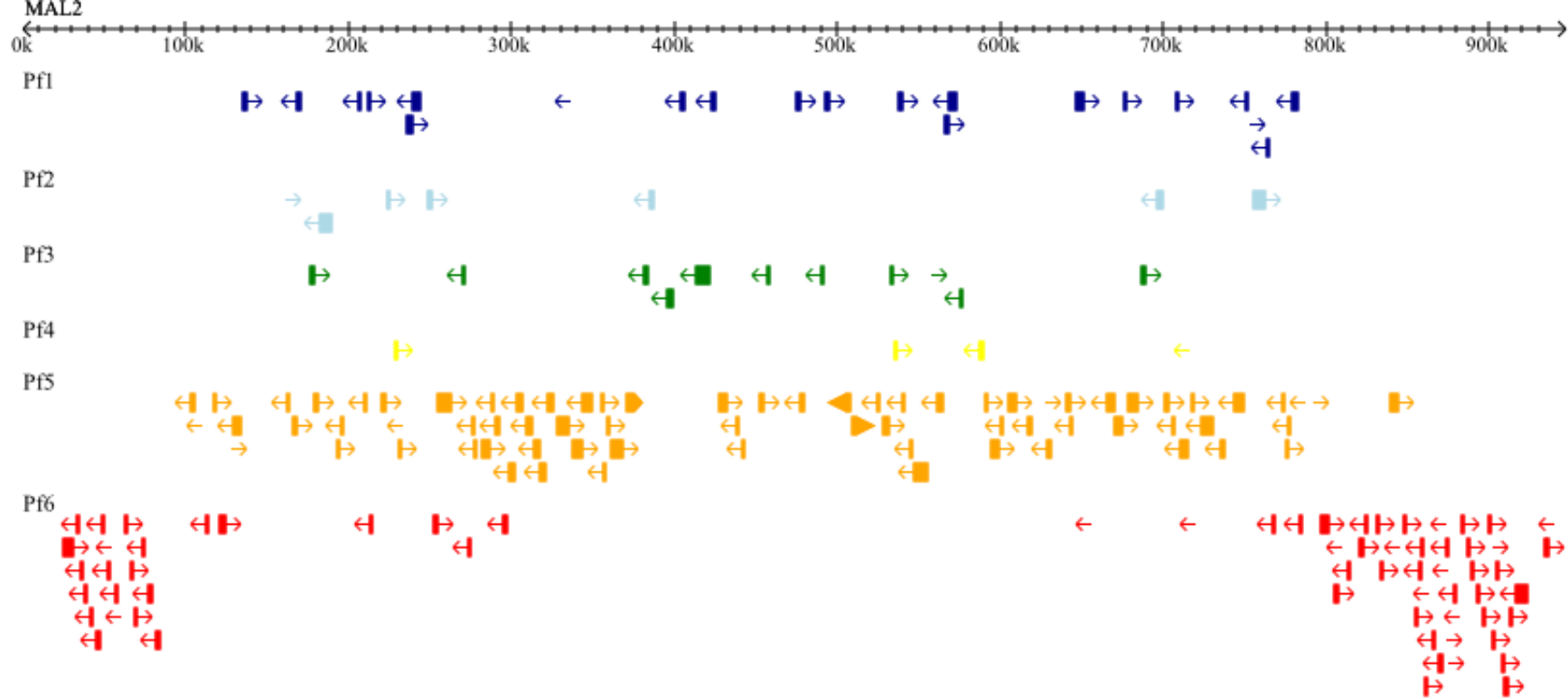

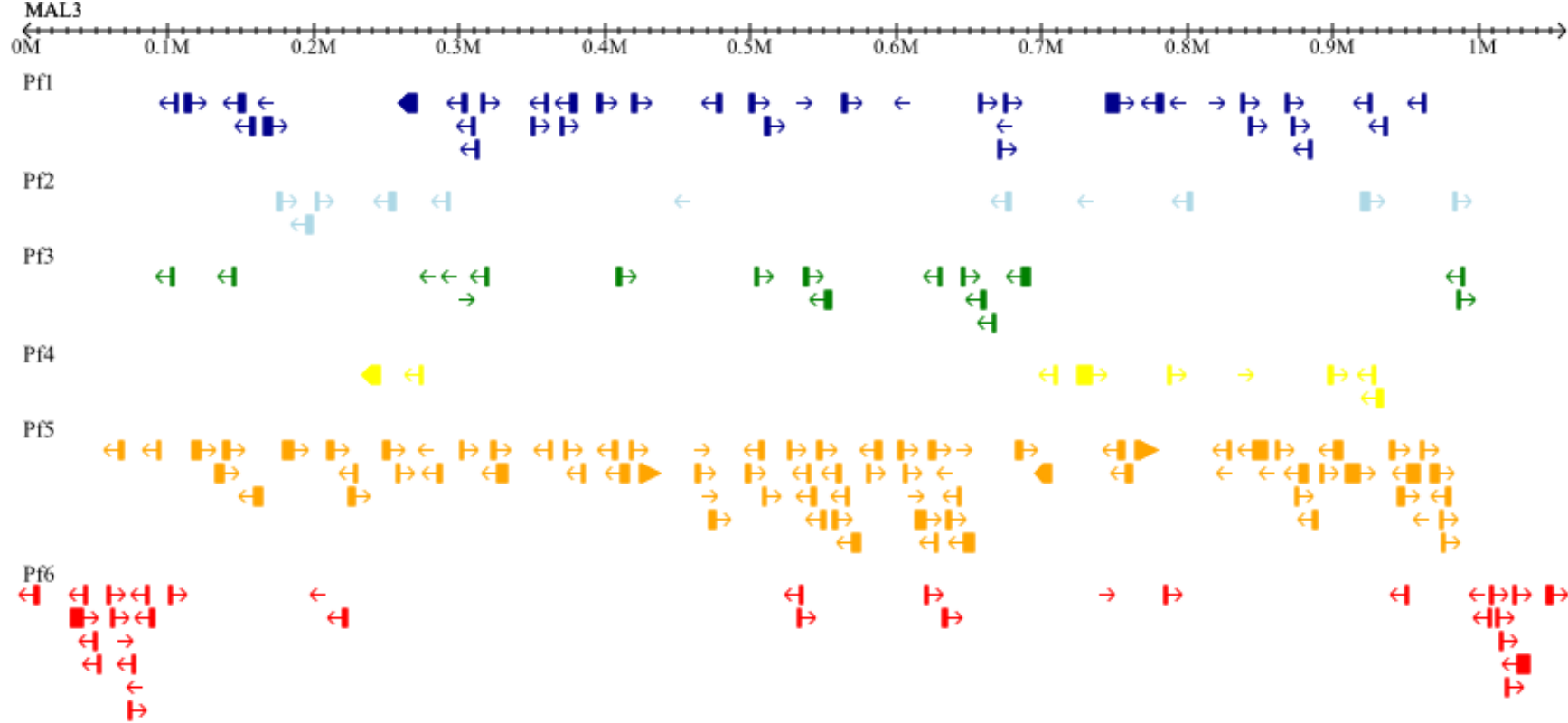

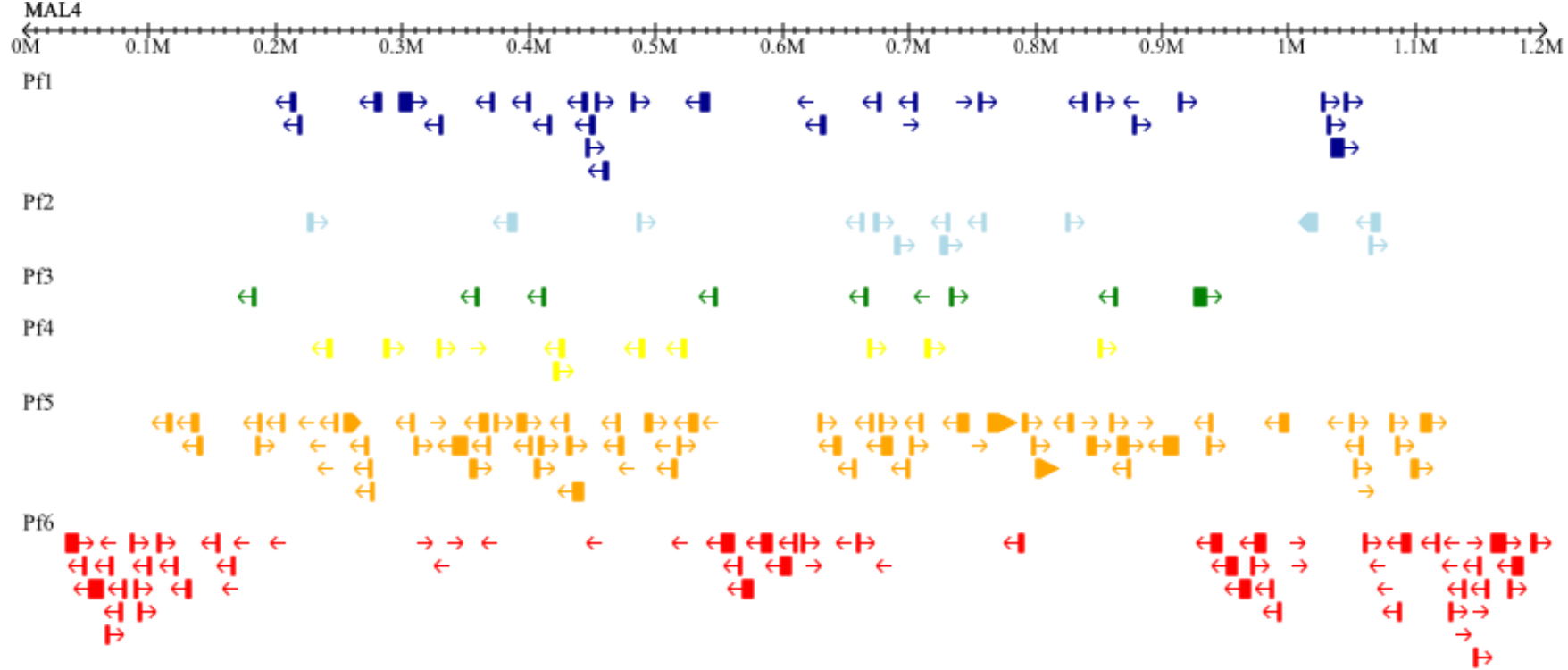

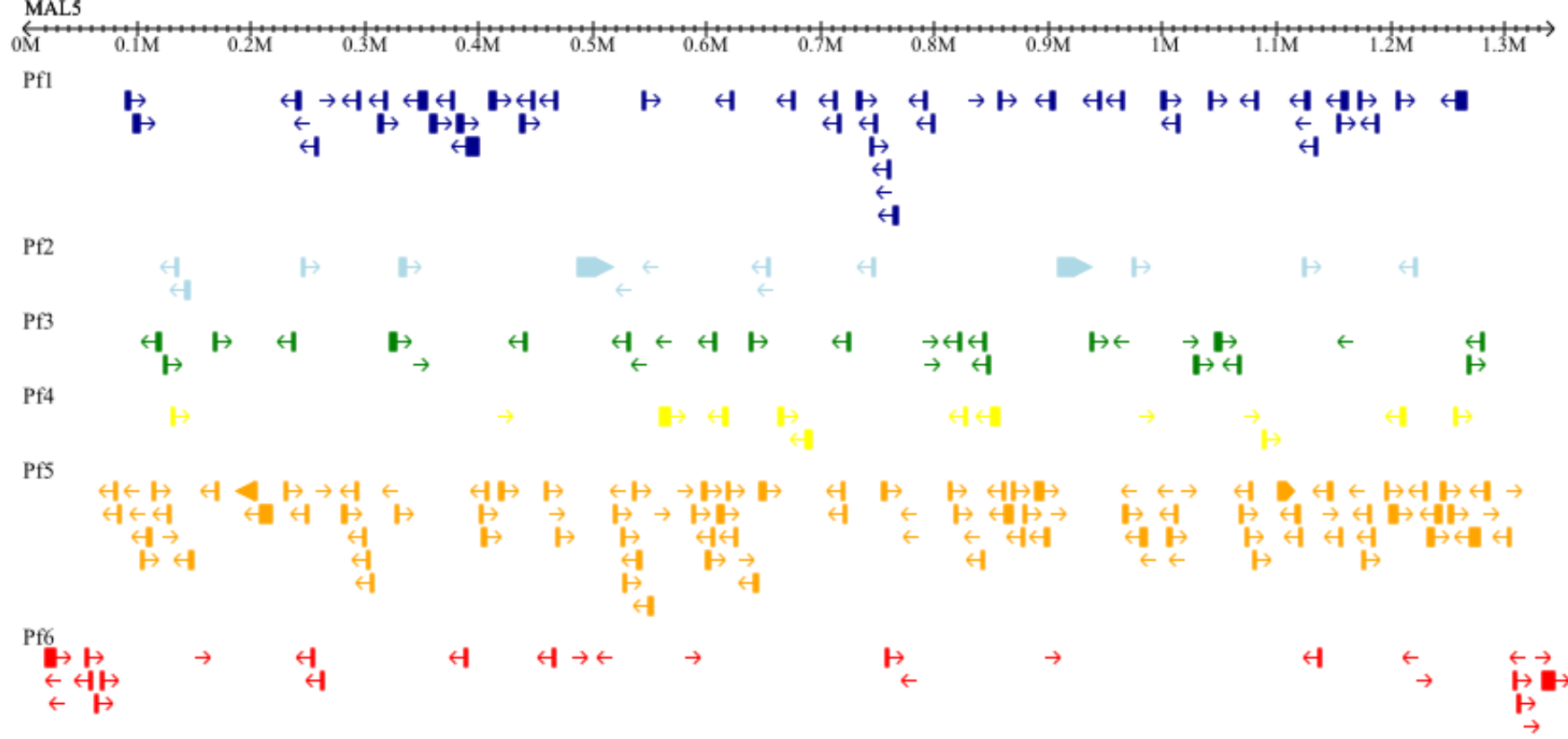

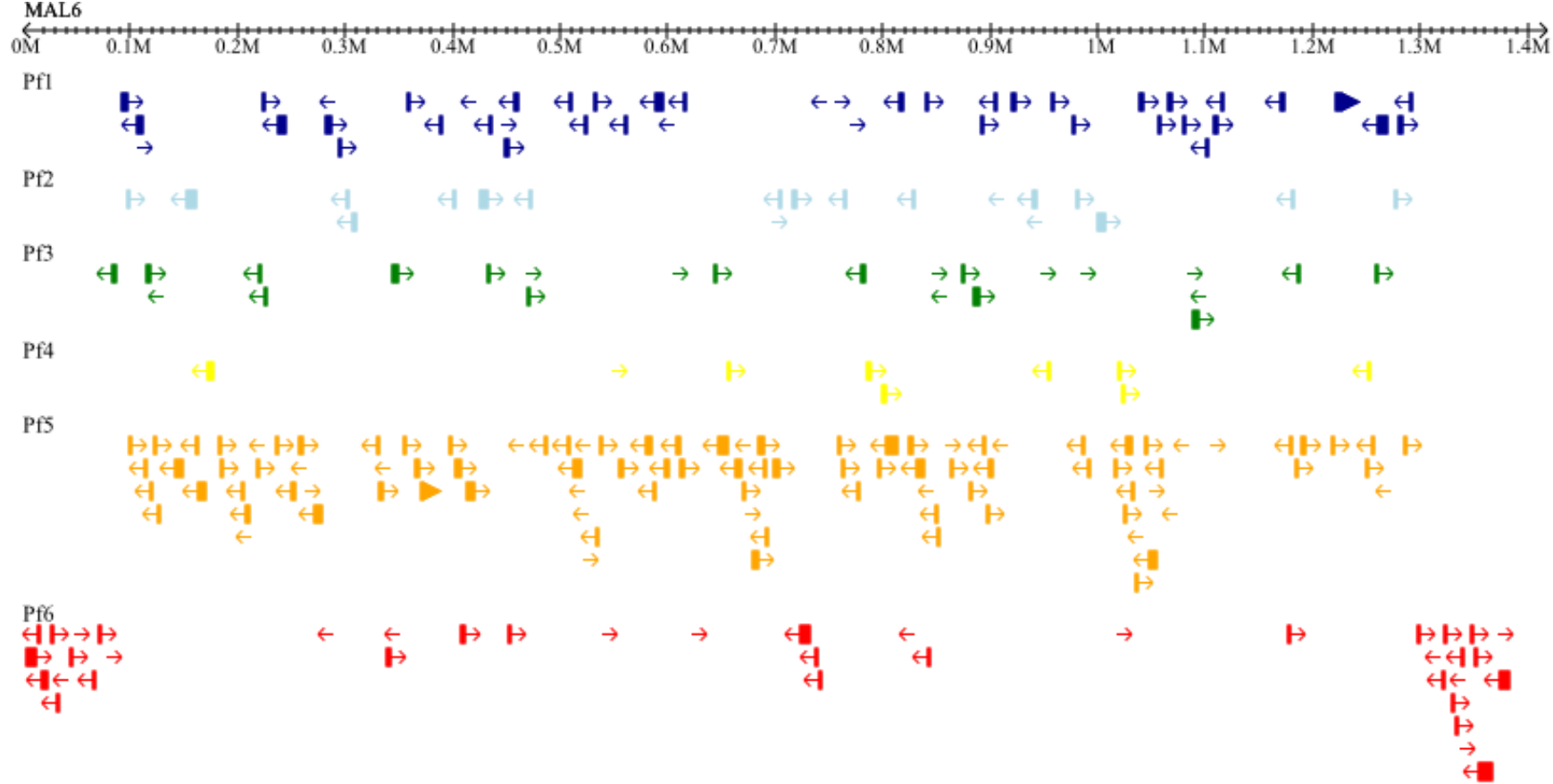

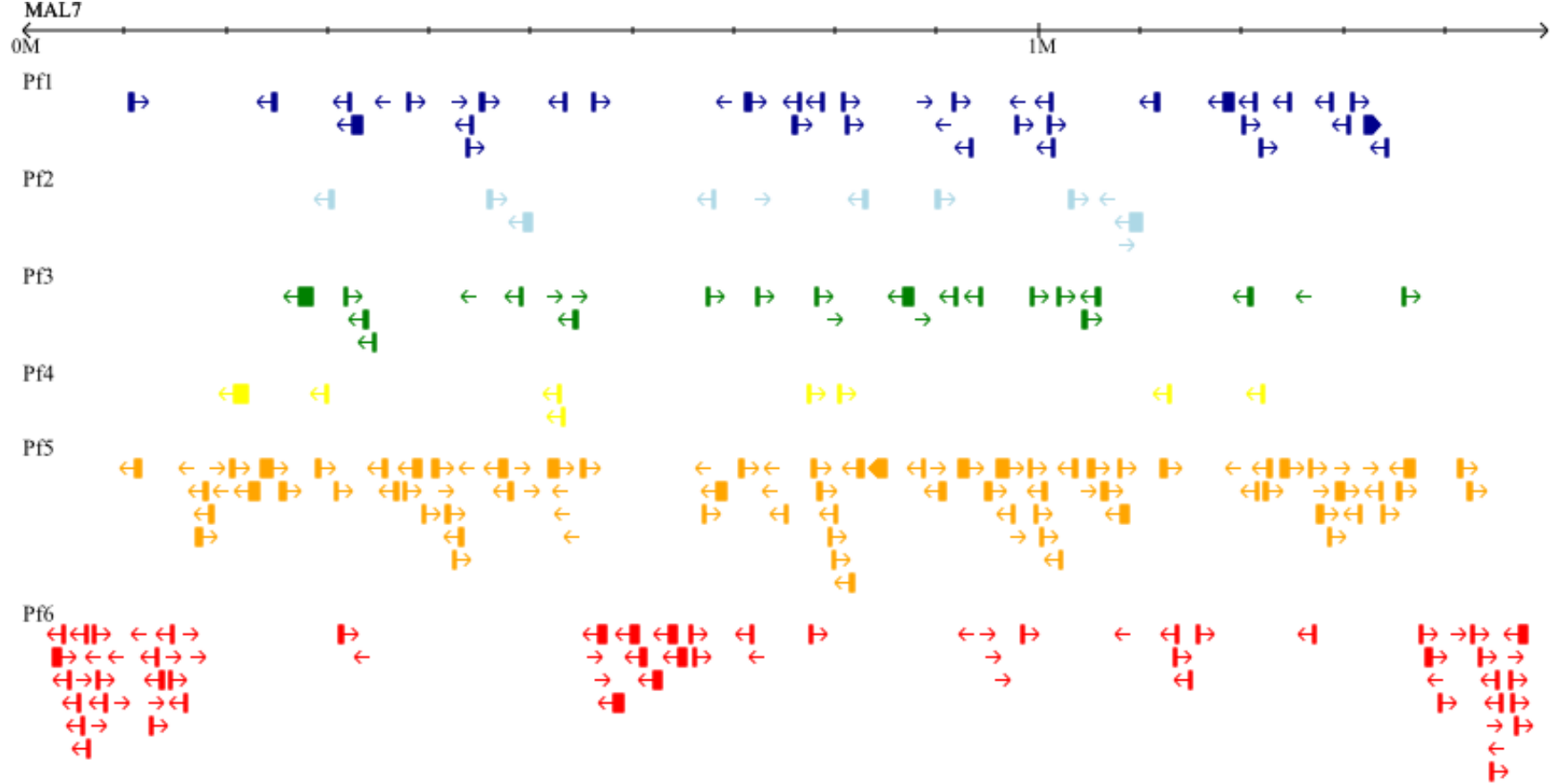

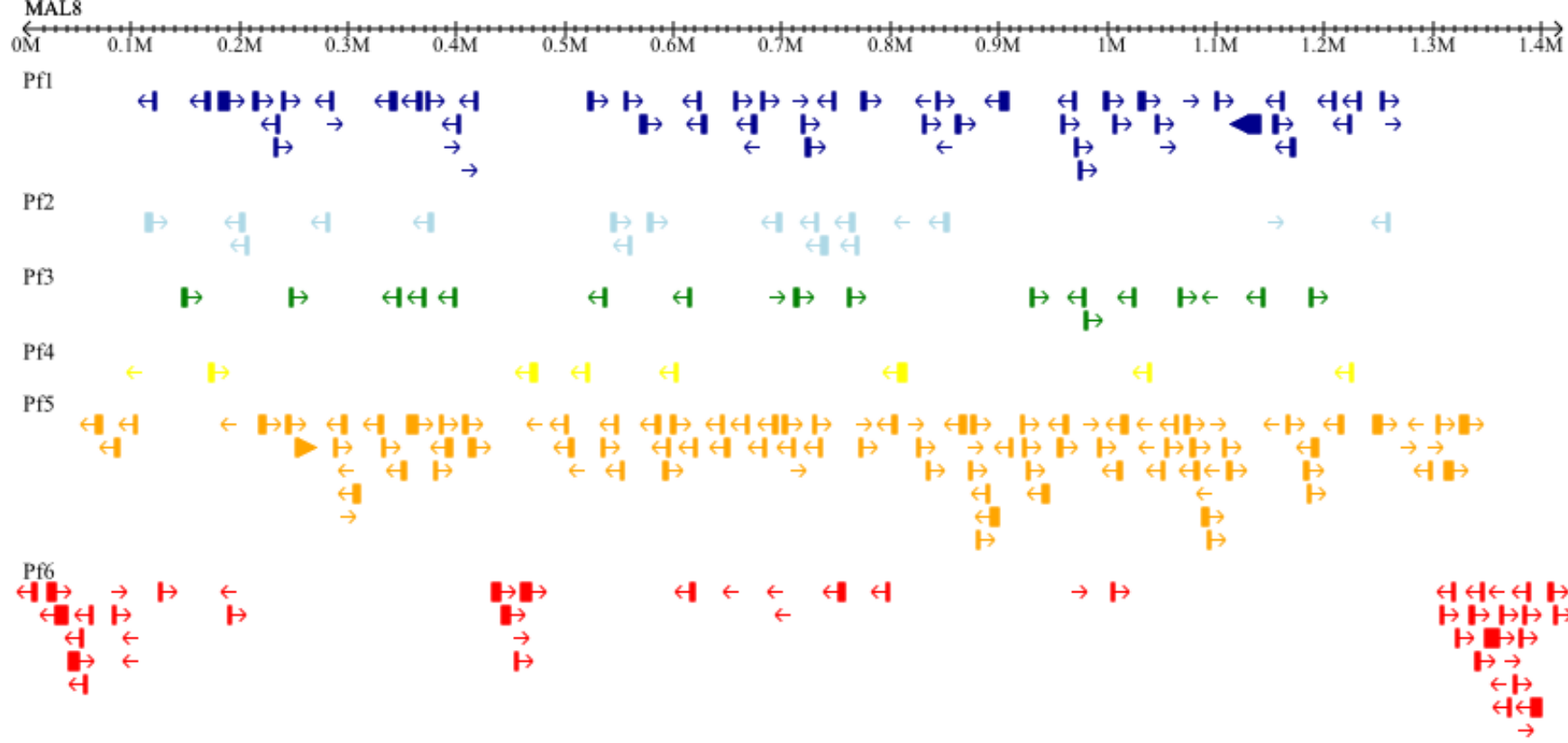

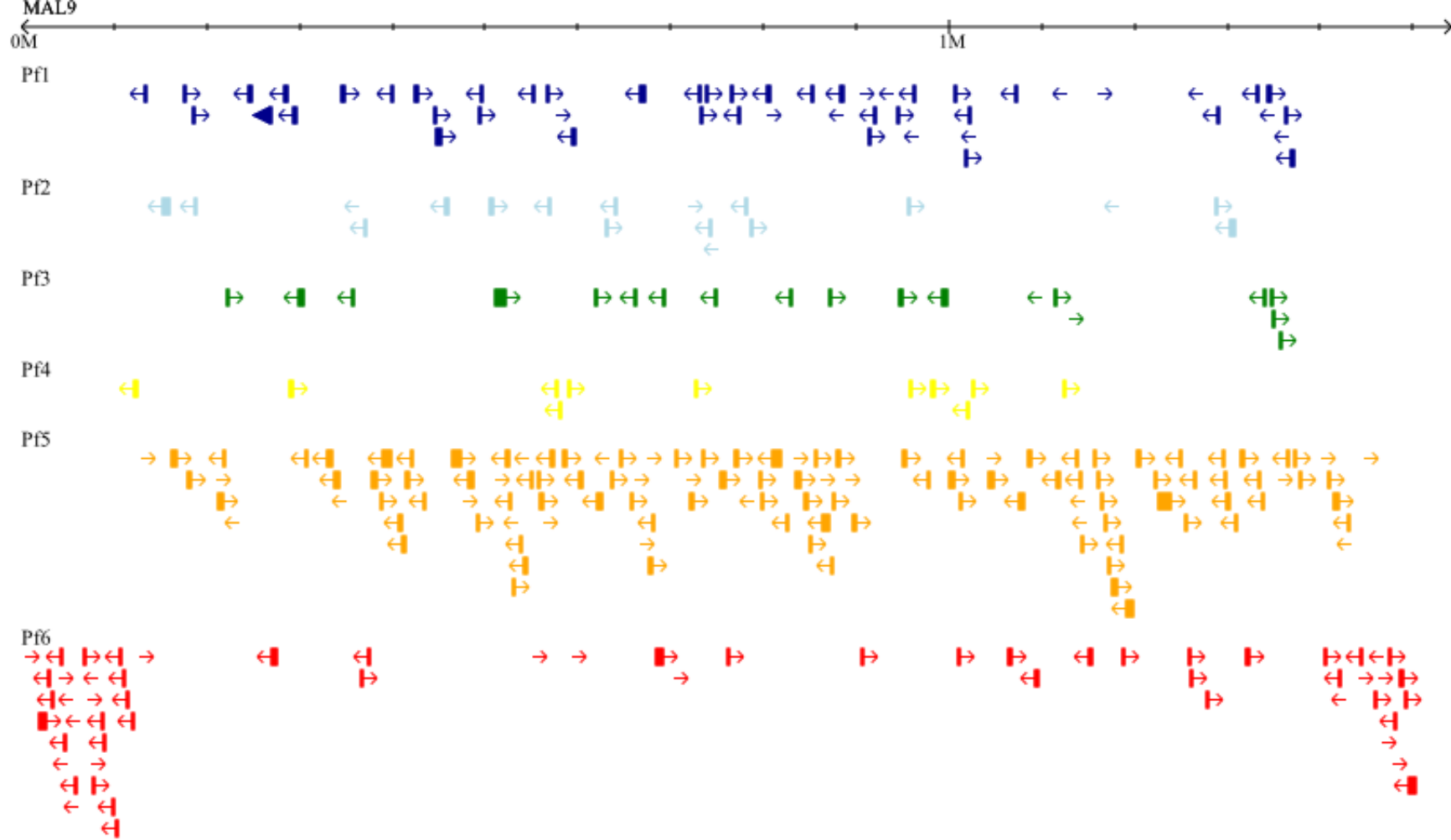

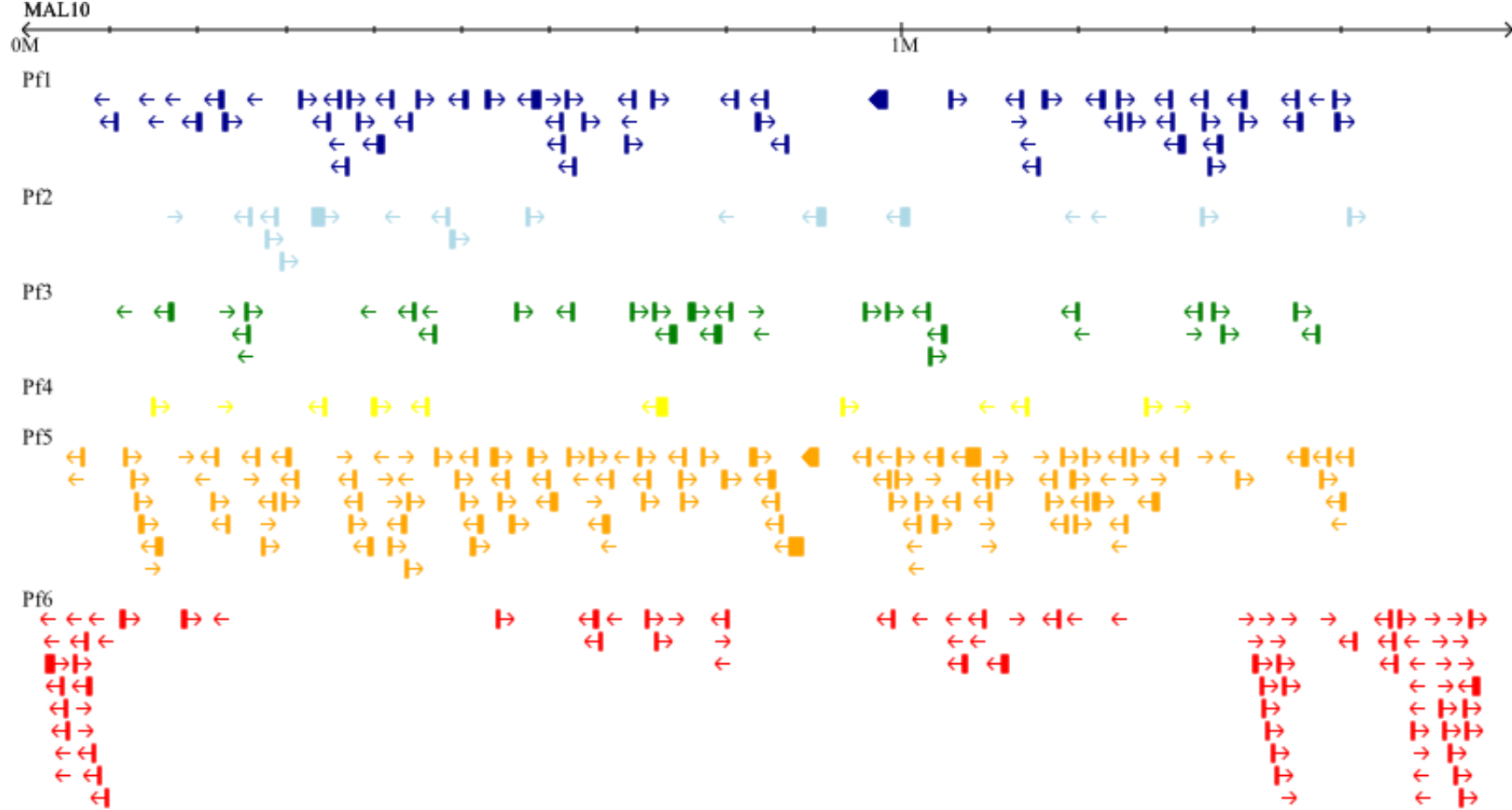

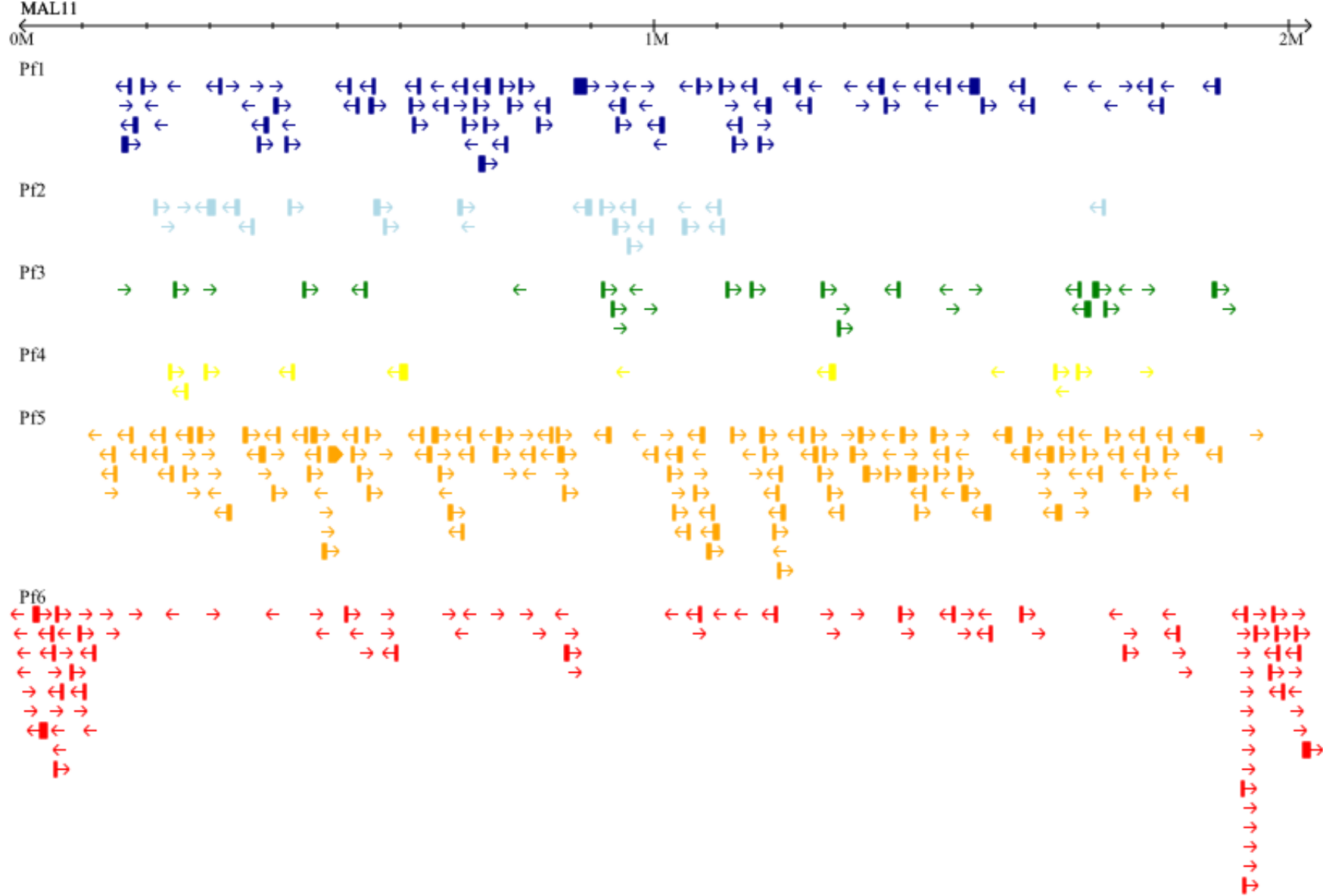

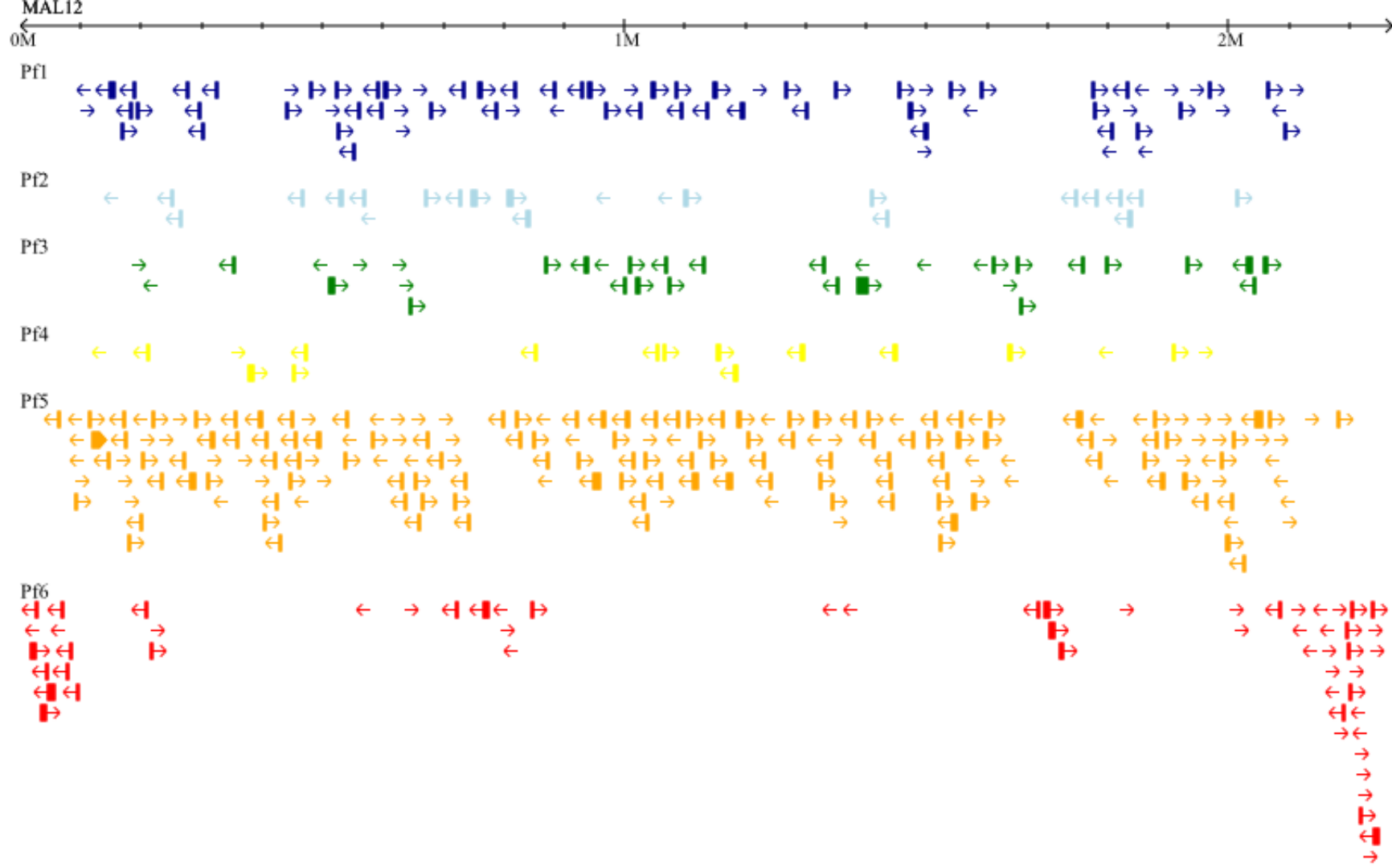

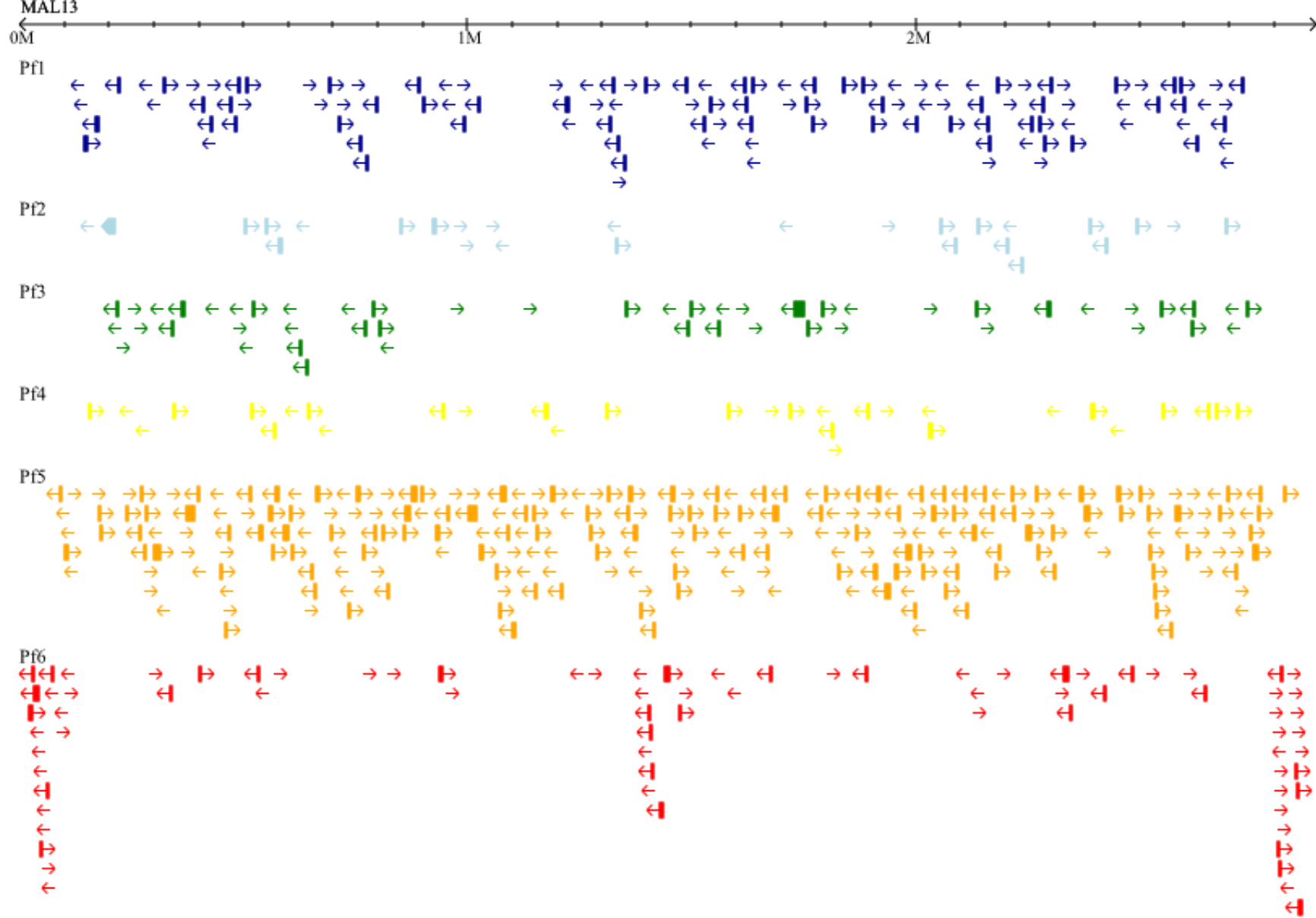

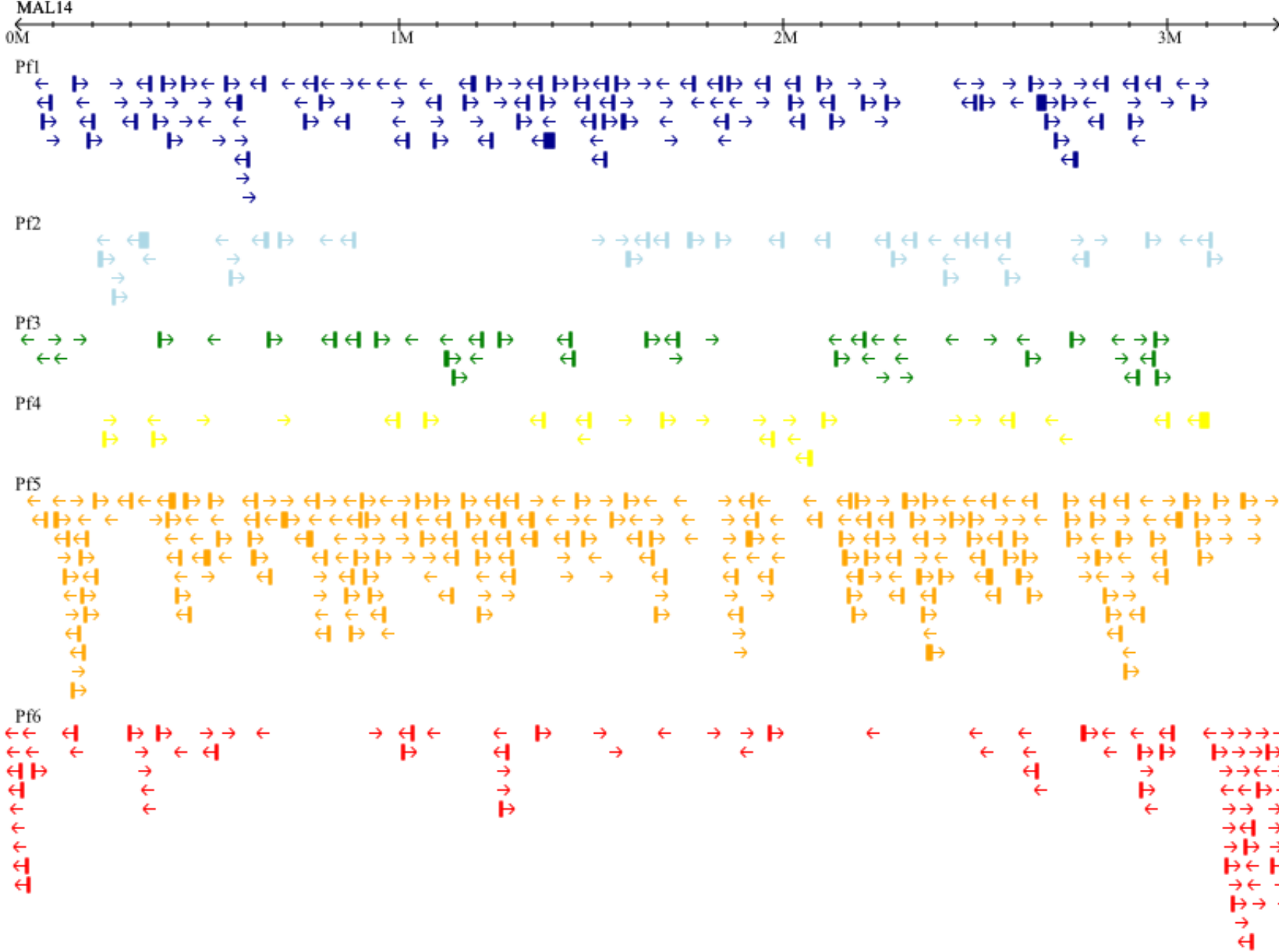

Supplement: Additional file 4 — Chromosomal location of lineage-specific genes in Plasmodium falciparum. Graphical distribution of genes on all 14 Plasmodium falciparum chromosomes. [file 1471-2148-8-108-S4.pdf]

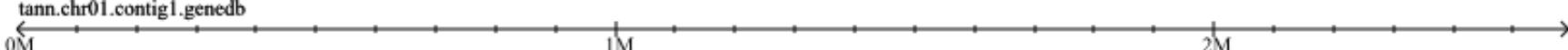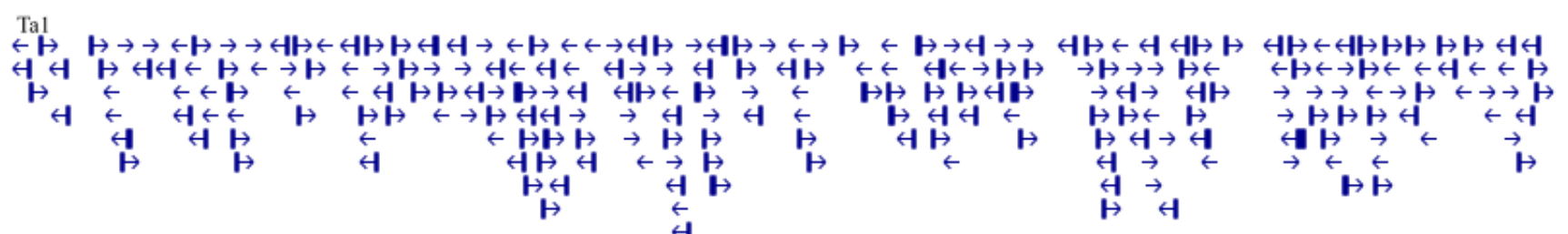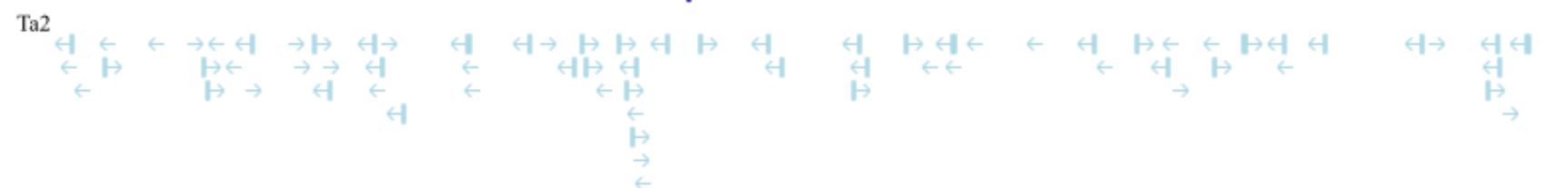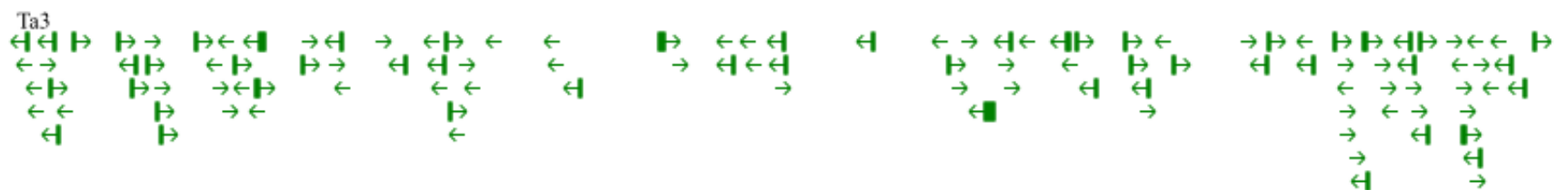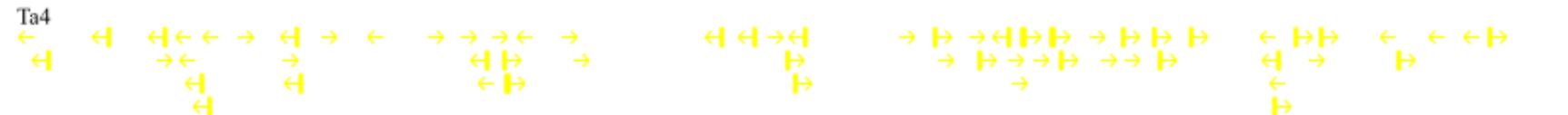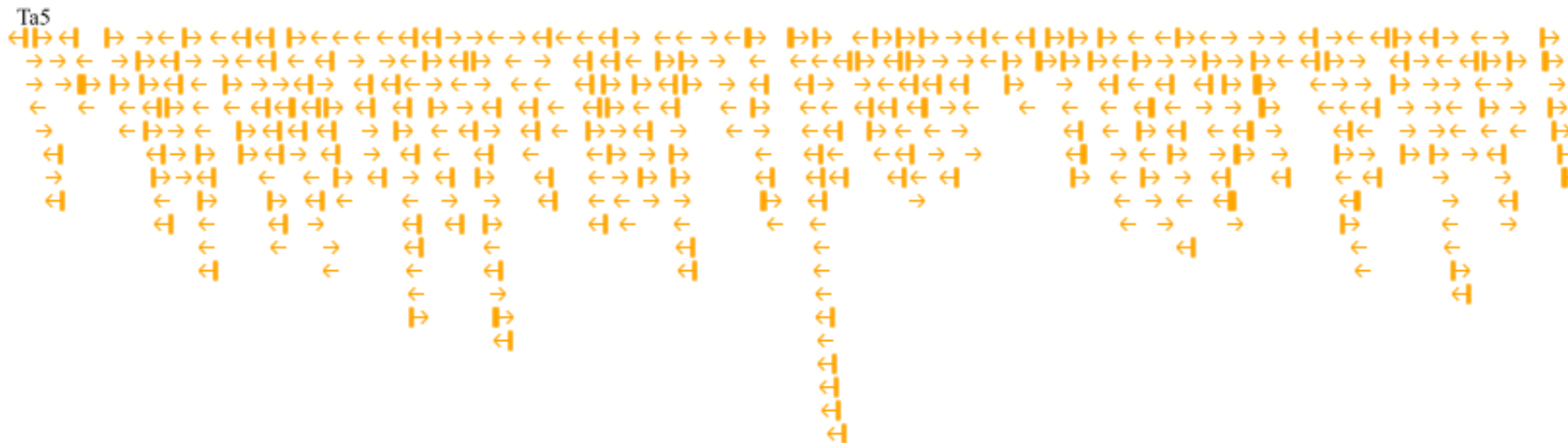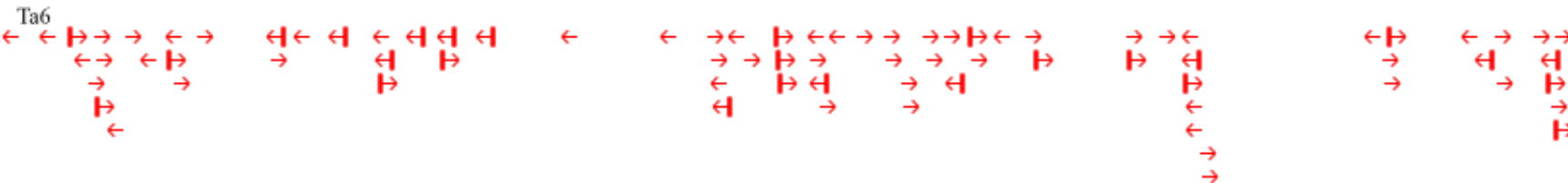

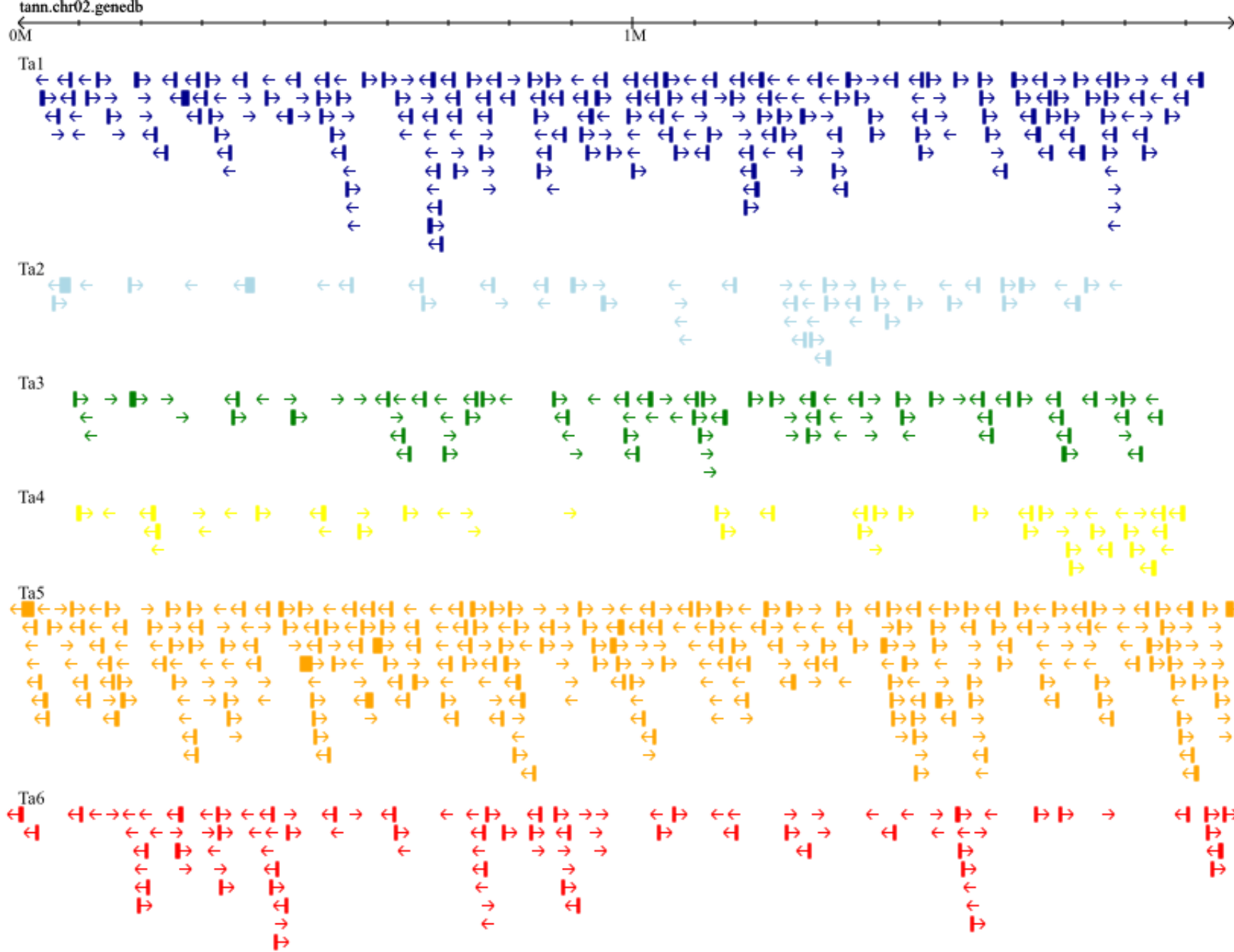

Ta1

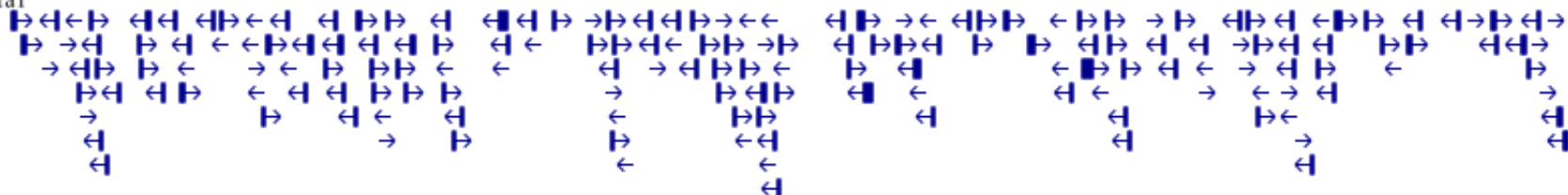

Ta2

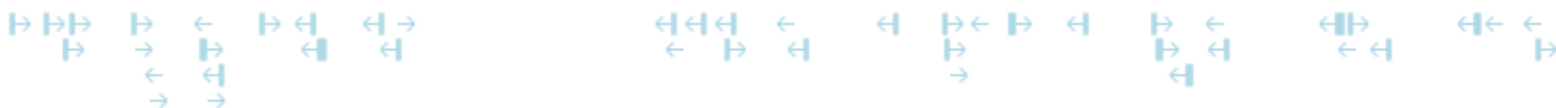

Ta3

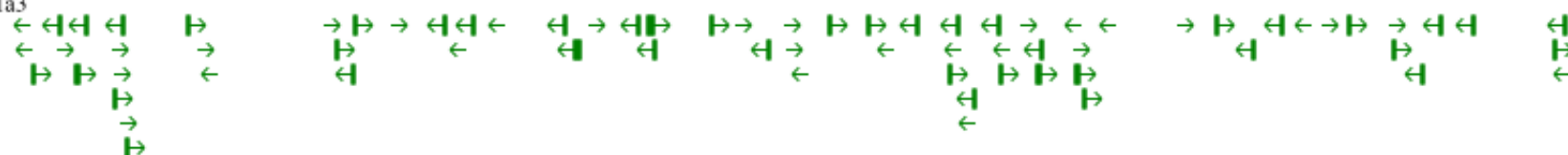

Ta4

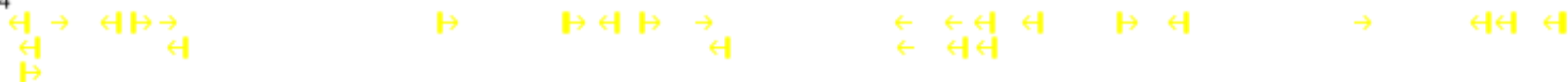

Ta5

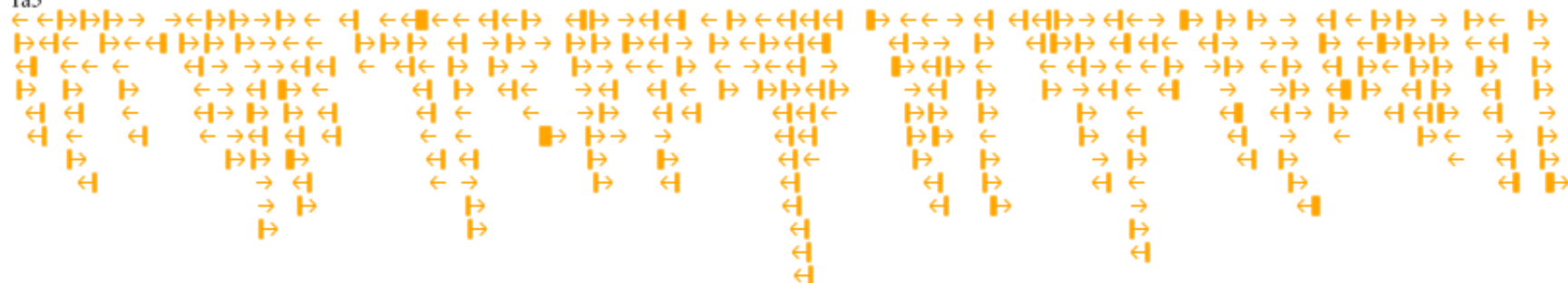

Ta6

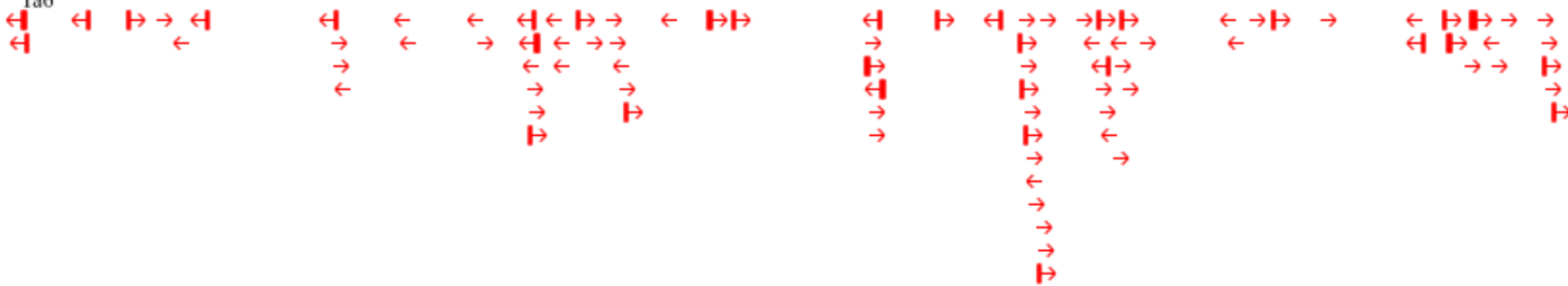

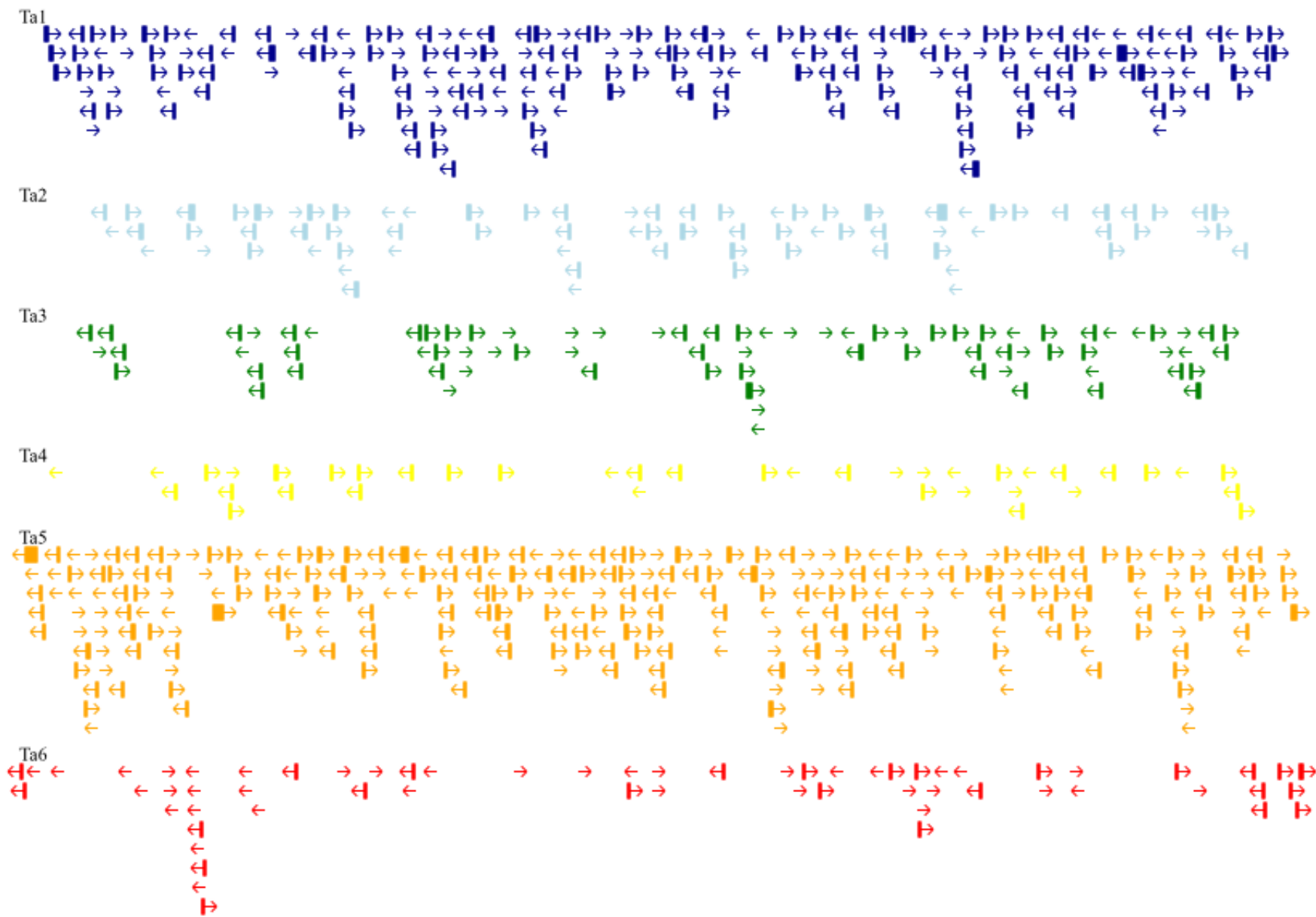

Supplement: Additional file 5 — Chromosomal location of lineage-specific genes in Theileria annulata. Graphical distribution of genes on all 4 Theileria annulata chromosomes. [file 1471-2148-8-108-S5.pdf]
